# The Regulatory Network of *CMPG1-V* in Wheat–*Blumeria graminis* f. sp. *tritici* Interaction Revealed by Temporal Profiling Using RNA-Seq

**DOI:** 10.3390/ijms21175967

**Published:** 2020-08-19

**Authors:** Jia Liu, Li Sun, Yiming Chen, Luyang Wei, Yongli Hao, Zhongyu Yu, Zongkuan Wang, Heng Zhang, Xu Zhang, Mengli Li, Haiyan Wang, Jin Xiao, Xiue Wang

**Affiliations:** State Key Laboratory of Crop Genetics and Germplasm Enhancement, Cytogenetics Institute, Nanjing Agricultural University/JCIC-MCP, Nanjing 210095, China; 2018201063@njau.edu.cn (J.L.); sunli@njau.edu.cn (L.S.); 2019101119@njau.edu.cn (Y.C.); 2019101121@njau.edu.cn (L.W.); haoyongli9128@163.com (Y.H.); 2019204044@njau.edu.cn (Z.Y.); wangzkuan@njau.edu.cn (Z.W.); 2016201031@njau.edu.cn (H.Z.); 2018201061@njau.edu.cn (X.Z.); limengli9393@163.com (M.L.); hywang@njau.edu.cn (H.W.); xiaojin@njau.edu.cn (J.X.)

**Keywords:** wheat powdery mildew, *Haynaldia villosa*, *CMPG1-V*, RNA-seq, hormone signaling, metabolism process

## Abstract

Wheat powdery mildew (Pm), caused by *Blumeria graminis* f. sp. *tritici* (*Bgt*), is a prevalent fungal disease. The diploid wheat relative *Haynaldia villosa* (*H. villosa*) showed broad-spectrum resistance (BSR) to Pm. A previous study reported an E3 ligase gene, *CMPG1-V* from *H. villosa*, showing BSR to Pm. To elucidate the regulatory network mediated by *CMPG1-V*, in this study, gene expression profiling of *CMPG1-V* transgenic plant (*CMPG1-V*_OE_) and its receptor Yangmai 158 was analyzed and compared after *Bgt* inoculation at four infection stages. GO and KEGG analysis revealed obvious reprogramming of SA and ABA signaling, starch/sucrose metabolism, and photosynthesis in *CMPG1-V*_OE_, compared with those in Yangmai 158. Transcripts of SA synthesis genes *SARD1* and *UGT*, signaling factors *TGA* and *PRs*, and *SnRKs* in ABA signaling were specifically upregulated in *CMPG1-V*_OE_ rather than Yangmai 158. Transcripts of *LHCII* in photosynthesis, *GLUC* and *TPP* in starch/sucrose metabolism were also induced distinctly in *CMPG1-V*_OE_. WGCNA analysis showed crucial regulatory candidates of *CMPG1-V*, involving serine/threonine-protein kinase in phosphorylation, glucosyltransferase in flavonoid biosynthesis, defense factor WRKYs, and peroxidase in oxidative stress. Our results facilitate the deciphering of the resistant regulatory network of *CMPG1-V* and the identification of key candidates which might be employed in breeding programs.

## 1. Introduction

When pathogens infect plants, the early defense signals mainly involve the hormone signaling pathways and oxidative homeostasis [1]. Ubiquitination regulated by E3 ubiquitin ligase plays an important role during the process [2]. SCF^COI1^ type E3 ubiquitin ligase subunit F-box protein COI1 (Coronatine insensitive 1) is the first confirmed ubiquitination system against pathogen infection in JA signaling [3]. Arabidopsis (*Arabidopsis thaliana*) RING E3 ubiquitin ligase Keep On Going (KEG) interacting with SCF^COI1^ E3 ubiquitin ligase positively regulates plant immunity [4]. A tobacco (*Nicotiana benthamiana*) RING-type E3 ubiquitin ligase, NtRFP1, has been shown recently to attenuate disease symptoms caused by bC1 protein through the ubiquitination of bC1 [5].

Salicylic acid (SA), considered to be an important endogenous signaling molecule in plant immunity, can promote the degradation of defense transcription repressors through E3 ubiquitin ligase. SA can promote E3 ubiquitin ligase BTB^NPR1^ degrading transcriptional repressors TGA2 and NIMIN1 to enhance disease resistance [6]. CUL4-DDB1-based ubiquitin ligase interacted with SA to enhance tomato’s resistance to nontumorigenic *Agrobacterium tumefaciens* [7]. AtSR1 interaction protein 1 (SR1IP1), a CUL3-based E3 ubiquitin ligase, positively regulates SA-related immunity by degrading the defense suppressor AtSR1 in Arabidopsis [8]. Several studies also confirmed the role of E3 ligases in ABA defense signaling. Overexpressing *OsDRF1* in tobacco, an F-box type E3 ligase in rice, showed ABA sensitivity and increased resistance to viruses and bacteria [9]. Pepper (*Capsicum annuum*) RING E3 ubiquitin ligase CaRING1 induced by *Xanthomonas oryzae* acts as a positive regulator of defense responses [10,11].

Ubiquitin ligase also regulates plant primary metabolism after pathogen infection. Ubiquitin ligase ARABIDOPSIS TOXICOS EN LEVADURA31 (ATL31) and its closest homologue, ATL6, a membrane-associated ubiquitin ligase, are involved in the carbon/nitrogen (C/N) response by regulating the stability of 14-3-3 proteins through ubiquitination in plant immune response [12,13]. Overexpression of *LeATL31* and *LeATL6* enhanced the *Pseudomonas syringae* resistance in tomato [14].

Wheat (*Triticum aestivum*, AABBDD) makes a substantial contribution to human calorie intake, global agricultural sustainability, and food security [15]. Pathogens and pests cause wheat yield losses that account for approximately 21.5% of total losses, reaching up to 28.1% in food-deficit areas [16]. Wheat powdery mildew (Pm), a fungal disease caused by *Blumeria graminis* f. sp. *tritici* (*Bgt*), is one of the most destructive diseases of wheat, causing extensive yield losses worldwide [17]. Due to the host or race specificity of the wheat–*Bgt* interaction, new virulent races could rapidly evolve and emerge. Thus, the exploration and utilization of durable and broad-spectrum resistance (BSR) genes are of great significance for powdery mildew control.

Although there have many studies on the resistance mechanism of ubiquitin ligase genes, only a few studies performed the transcriptomic changes in response to Pm in a continuous time period. The related metabolism reprogramming in the disease resistance process is also indistinct. In a previous study, an E3 ligase gene, *CMPG1-V*, was cloned from *Haynaldia villosa*. Overexpression of *CMPG1-V* enhanced its BSR to Pm [18]. This provides an ideal system for investigating the temporal process during *Bgt* infection regulated by *CMPG1-V*. In this study, *CMPG1-V* transgenic line (*CMPG1-V*_OE_) and susceptible receptor Yangmai 158 were inoculated with *Bgt* isolate E26 for RNA-seq. *CMPG1-V* associated defense pathways were analyzed by the Gene Ontology (GO), Kyoto Encyclopedia of Genes and Genomes (KEGG) enrichment, and Weighted Gene Co-Expression Network (WGCNA). These findings provide insights into the molecular mechanisms of *CMPG1-V*-associated broad-spectrum powdery mildew resistance in wheat.

## 2. Results

### 2.1. Global Profiling of CMPG1-V_OE_ in Response to Bgt Infection

**Figure 1:**
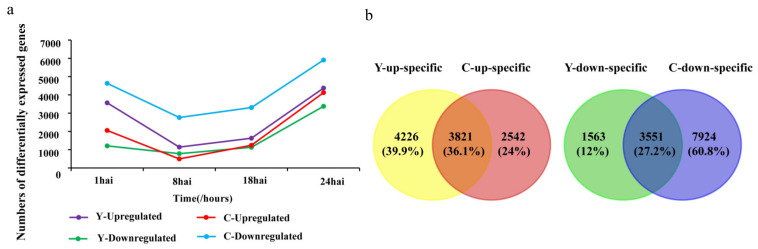
Differentially expressed genes (DEGs) in *CMPG1-V*_OE_ and susceptible receptor Yangmai 158 after *Bgt* infection. (**a**) DEGs of *CMPG1-V*_OE_ and Yangmai 158 in different infection points. Purple and green represent upregulated and downregulated DEGs in Yangmai 158, red and blue represent upregulated and downregulated DEGs in *CMPG1-V*_OE_, respectively. (**b**) Venn diagram of upregulated and downregulated DEGs in *CMPG1-V*_OE_ and Yangmai 158. (FDR ≤ 0.001; genes with the regulation ratio log ≥2 or ≤−2 were selected).

When *Bgt* infects wheat, conidia forms rapidly and reaches the epidermal cells from 1 h after inoculation (hai) to 72 hai (Figure S1) [19]. Based on the above observation, the samples for RNA-seq of *CMPG1-V* transgenic line (*CMPG1-V*_OE_) and susceptible receptor Yangmai 158 were collected at 1, 8, 18, and 24 hai by moderately *Bgt* virulent race E26. The mock samples were collected simultaneously at the above corresponding time points. The Q20 and Q30 of RNA-seq sequences for all samples were more than 93% and 84% and the ratios of clean reads accounted for 89.41% to 95.17%, respectively (Table S1). To maximize the number of possible differentially expressed genes (DEGs), the selective parameter was set as Fold Change ≥ 2.00 and FDR ≤ 0.001. In *CMPG1-V*_OE_ and Yangmai 158, 6363 and 8047 transcripts were upregulated, and 11,475 and 5114 were downregulated, respectively (Figure 1a). The number of upregulated DEGs in *CMPG1-V*_OE_ was less than that in Yangmai 158 for all the analyzed infection time points. Venn diagram revealed 24% of the upregulated DEGs were specifically expressed in *CMPG1-V*_OE_, while for the downregulated DEGs, up to 60.8% were specifically expressed in *CMPG1-V*_OE_, and only 12% were specifically expressed in Yangmai 158 (Figure 1b). We have also tested the two materials with a more virulent race, E31, and harvested the samples for RNA-seq at the same time points after inoculation (Table S1 and Figure S2).

### 2.2. Functional Categories Enriched in CMPG1-V_OE_ and Yangmai 158 in Response to Bgt Infection

To understand the biological function of the DEGs, GO enrichment was performed to classify the crucial biological processes and cardinal signaling pathways. Before *Bgt* infection, no significant difference was observed between *CMPG1-V*_OE_ and Yangmai 158 (Figure S3a). The upregulated DEGs in *CMPG1-V*_OE_ were enriched in normal physiological metabolism, such as “sulfur compound transport”, “aminoglycan metabolic process”, “glutathione metabolic process”, “oxoacid metabolic process”, and so on.

**Figure 2:**
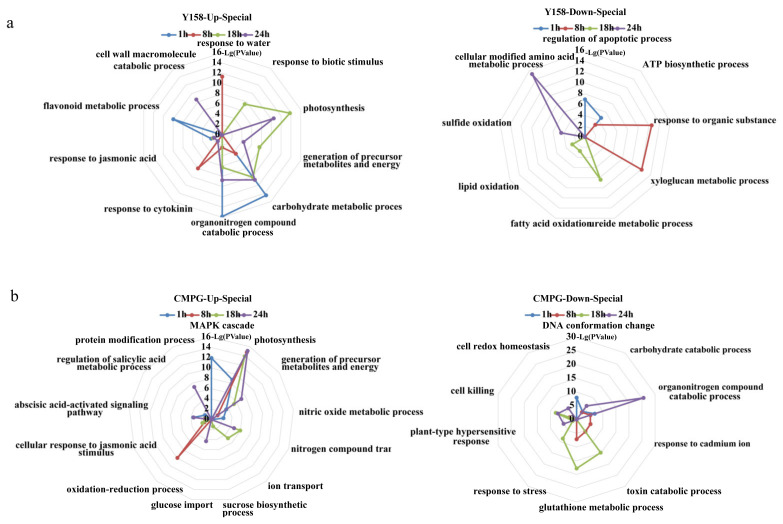
Specificity categories in susceptible receptor Yangmai 158 (**a**) and *CMPG1-V*_OE_ (**b**) after *Bgt* infection. Upregulated and downregulated DEGs from 1 hai to 24 hai were selected as follows: *P* value < 0.05; genes with the regulation ratio log ≥ 2 or ≤−2 were selected.

After *Bgt* inoculation, we observed distinct enriched DEGs in the two materials (Figure 2a,b). At 1 hai, most of the upregulated DEGs in the two materials focused on “photosynthesis” and “generation of precursor metabolites and energy”, and transcription levels increased in the succeeding infection timepoints. At later infection stages from 18 hai to 24 hai, the upregulated transcripts in “abscisic acid (ABA)-activated signaling pathway”, “regulation of salicylic acid (SA) metabolic process”, “glucose import and sucrose biosynthetic process”, “nitrogen compound”, and “nitric oxide metabolic process” were specifically enriched in *CMPG1-V*_OE_ (Figure 2b). However, at the corresponding infectious timepoints in Yangmai 158, the upregulated transcripts were only enriched in “organonitrogen compound catabolic process”, “response to cytokinin”, and “carbohydrate metabolic process” (Figure 2a). Similarly, the hormone and energy biological processes were also observed in *CMPG1-V*_OE_ challenged with E31 (Figure S4).

The special downregulated DEGs in *CMPG1-V*_OE_ were mainly enriched in “glutathione metabolic process”, “toxin catabolic process”, and “organonitrogen compound catabolic process” (Figure 2b), while the transcripts in “ATP biosynthetic process”, “response to organic substance”, “fatty acid and lipid oxidation”, and “sulfide oxidation” were specially downregulated in Yangmai 158 (Figure 2a and Figure S4).

Common up- or downregulated DEGs were also present in the two materials after *Bgt* inoculation. The upregulated transcripts of common defense response in *CMPG1-V*_OE_ and Yangmai 158 were enriched in “cell wall organization or biogenesis”, “glutathione metabolic process”, “toxin catabolic process”, and “sulfur compound transport” (Figure S3b). The downregulated transcripts were enriched in “carboxylic acid biosynthetic process”, “fatty acid biosynthetic process”, “response to acid chemical”, and “chitin metabolic process” (Figure S3c).

### 2.3. Temporal Specificity of CMPG1-V_OE_ in Response to Bgt Infection

**Figure 3:**
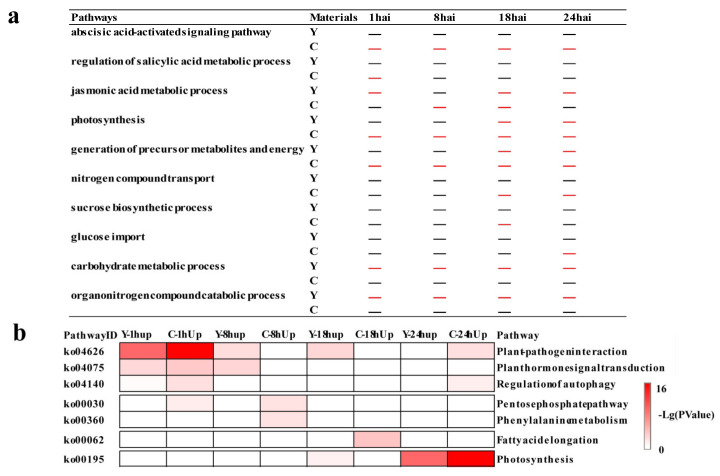
GO classification and KEGG pathways enrichment in *CMPG1-V*_OE_ and susceptible receptor Yangmai 158 from 1 hai to 24 hai after *Bgt* infection. (**a**) GO analysis of special upregulated genes in Yangmai 158 and *CMPG1-V*_OE_ from 1 hai to 24 hai. (**b**) Heat map showing the *P*-value significance of enriched KEGG pathways of upregulated DEGs in *CMPG1-V*_OE_ and Yangmai 158 from 1 hai to 24 hai. Y represents Yangmai 158, C represents *CMPG1-V*_OE_. The colors white, pink, and red represent low, medium, and high expression levels, respectively. Upregulated pathways were selected as follows: *P* value < 0.05; genes with the regulation ratio log ≥ 2 or ≤−2 were selected.

To characterize the pathways regulated by *CMPG1-V* at different time points, temporal specificity of *CMPG1-V*_OE_ was analyzed (Figure 3a). At 1 hai, “ABA and SA pathways” firstly appeared in *CMPG1-V*_OE_. SA signaling pathway was only present in *CMPG1-V*_OE_ at 1 hai, and ABA-associated processes were sustained during all the tested time points in *CMPG1-V*_OE_. “Jasmonic acid (JA) pathway” was induced in *CMPG1-V*_OE_ at 8 hai, while it was induced at 1 hai and sustained from 18 to 24 hai in Yangmai 158 after *Bgt* infection. Moreover, transcripts in “photosynthesis” and “generation of precursor metabolites and energy” were significantly upregulated in *CMPG1-V*_OE_ at 1 hai and thereafter. From 18 hai to 24 hai, the “nitrogen compound transport” was specifically enriched in *CMPG1-V*_OE_. The “sucrose biosynthetic process” and “glucose import” were enriched specifically in *CMPG1-V*_OE_ at 18 hai and 24 hai, respectively. In Yangmai 158 at 18 and 24 hai, there was no significant enrichment of the above three pathways, but “photosynthesis” and “generation of precursor metabolites and energy” were specifically enriched. The “carbohydrate metabolic process” and “organonitrogen compound catabolic process” were activated in Yangmai 158, while not in *CMPG1-V*_OE_ from 1 hai to 24 hai.

KEGG enrichment analysis was used to classify the crucial biological processes and cardinal signaling pathways (Figure 3b). At 1 hai, the “plant–pathogen interaction” and “plant hormone signal transduction” were obviously upregulated in *CMPG1-V*_OE_, and the transcription level in “regulation of autophagy” increased twofold more in *CMPG1-V*_OE_ than in Yangmai 158. At 8 hai and the later infection stages, the “phenylalanine metabolism” was activated and increased up to threefold more in *CMPG1-V*_OE_ than in Yangmai 158. Metabolic processes such as “fatty acid elongation” increased more than twofold in *CMPG1-V*_OE_. With the infection going on, “photosynthesis” was activated both in *CMPG1-V*_OE_ and Yangmai 158 at 24 hai, but more significantly in *CMPG1-V*_OE_. The downregulated DEGs were mainly enriched in “glycine, serine, and threonine metabolism”, “ABC transporters”, “isoflavonoid biosynthesis”, and “glutathione metabolism” (Figures S5 and S6).

### 2.4. Phytohormone Signaling Was Rapidly Reprogrammed in CMPG1-V_OE_ after Bgt Infection

**Figure 4:**
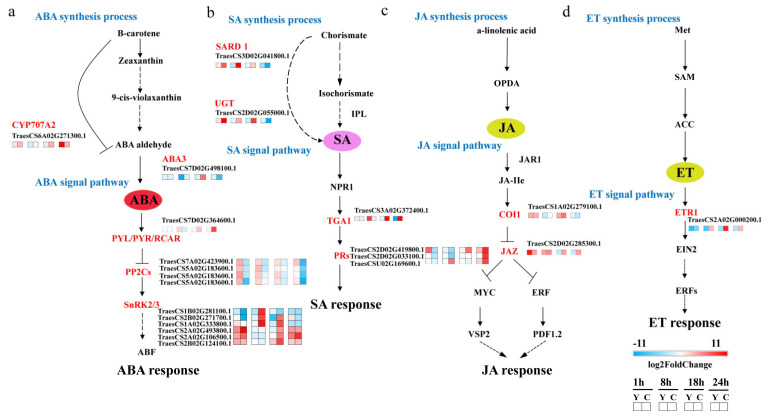
Differentially expressed genes related to plant hormone pathways in *CMPG1-V*_OE_ and susceptible receptor Yangmai 158 from 1 hai to 24 hai by *Bgt* infection. (**a**) ABA pathway; (**b**) SA pathway; (**c**) JA pathway; (**d**) ET pathway. Heat map showing the gene expression. The colors blue, white, and red represent low, medium, and high expression levels, respectively. Y represents Yangmai 158, C represents *CMPG1-V*_OE_. Genes with the regulation ratio log ≥ 2 or ≤−2 were selected.

**Figure 5:**
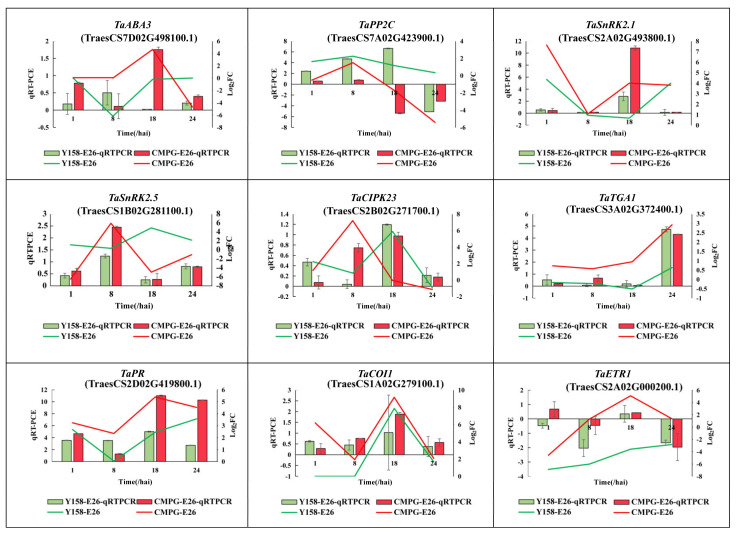
qRT-PCR and sequencing data of genes expression in *CMPG1-V*_OE_ and susceptible receptor Yangmai 158 from 1 hai to 24 hai after *Bgt* infection. The histogram represents qRT-PCR of genes expression. Green represents Yangmai 158, red represents *CMPG1-V*_OE_. Line chart represents sequencing data of genes expression. The green line represents Yangmai 158, red line represents *CMPG1-V*_OE_.

Transcription analysis indicated obvious biosynthesis and signaling changes in ABA and SA pathways in *CMPG1-V*_OE_ in response to E26 infection. For the ABA pathway, its degradation gene *CYP707A2* decreased more than threefold, while its synthesis gene *ABA3* increased more than 4.5-fold at 18 hai in *CMPG1-V*_OE_ (Figure 4a). More, *PP2C*, a negative regulatory gene in ABA signaling, was downregulated in *CMPG1-V*_OE_ for all the analyzed infection processes, while transcripts of *SnRK2* and *SnRK3* were activated evidently in *CMPG1-V*_OE_ in all infectious time points (Figure 4a). *SnRK2.1* increased more than twofold and *SnRK2.5* increased more than fivefold in *CMPG1-V*_OE_ than in Yangmai 158. Besides, *CIPK23* (*SnRK3*) increased more than sevenfold. The qRT-PCR also validated the RNA-seq data. As shown in Figure 5, the expression level of synthesis gene *ABA3* peaked at 18 hai in *CMPG1-V*_OE_, not in Yangmai 158; *PP2C* decreased in *CMPG1-V*_OE_ from 18 hai to 24 hai, while *SnRK2.1* increased to the maximum at 18 hai and *SnRK2.5* peaked at 8 hai in *CMPG1-V*_OE_; and the expression level of *CIPK23* maximized at 8 hai in *CMPG1-V*_OE_, not in Yangmai 158.

For the SA pathway, SA synthesis and transduction pathway were distinctly more upregulated in *CMPG1-V*_OE_. Transcript levels of critical genes for SA synthesis, *SAR DEFICIENT1* (*SARD1*) and *UDP-glycosyltransferase* (*UGT*), were obviously increased in *CMPG1-V*_OE_ when infected by *Bgt*. The *UGT* was threefold higher in *CMPG1-V*_OE_ from 1 hai to 18 hai and *SARD1* was threefold more in *CMPG1-V*_OE_ than in Yangmai 158 from 1 hai to 18 hai (Figure 4b). The activation of the SA signal pathway was shown by the *TGA1* expression increasing more than sixfold in *CMPG1-V*_OE_ from 1 hai to 18 hai; the *PR* was more than fourfold in *CMPG1-V*_OE_ at 8 hai and increased to ninefold at 24 hai (Figure 4b), while no obvious induction of these genes was observed in Yangmai 158 (Figure 4b). qRT-PCR of *TaTGA1* and *TaPR1* validated the results from the RNA-seq data (Figure 5). The expression level of *TaTGA1* peaked at 24 hai in *CMPG1-V*_OE_ and *TaPR1* expression rose in *CMPG1-V*_OE_ from 18 hai to 24 hai, which were more than those in Yangmai 158.

In addition, transcription levels of *COI1* in JA signaling and *ETR1* in ET signaling were upregulated in *CMPG1-V*_OE_ (Figure 4c,d). The expression levels of *COI1* and *ETR1* maximized at 18 hai in *CMPG1-V*_OE_, which was more than those in Yangmai 158 (Figure 5). The upregulated hormone signaling and associated DEGs were also observed in *CMPG1-V*_OE_ or Yangmai 158 tested with E31 (Figure S7).

### 2.5. CMPG1-V Activates Conspicuous Energy Metabolic Signaling during Bgt Infection

**Figure 6:**
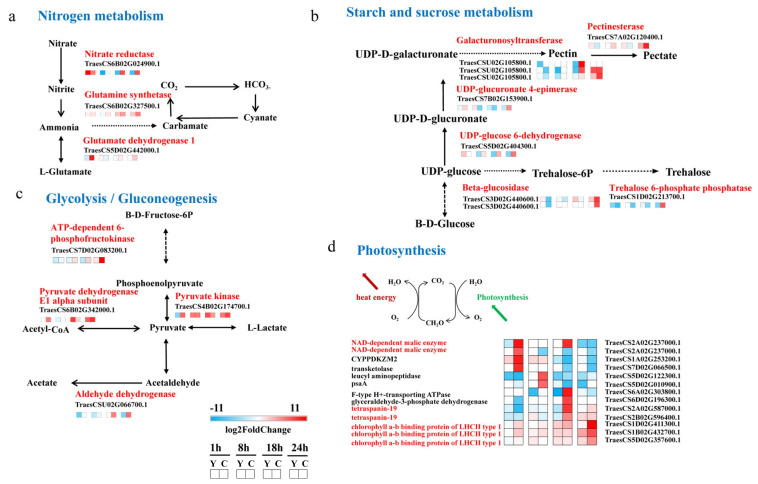
*CMPG1-V*_OE_ and Yangmai 158 DEGs related to four energy metabolic signaling pathways. (**a**) Nitrogen metabolism; (**b**) starch and sucrose metabolism; (**c**) glycolysis/gluconeogenesis; (**d**) photosynthesis. Heat map showing the gene expression. The colors blue, white, and red represent low, medium, and high expression levels, respectively. Y represents Yangmai 158, C represents *CMPG1-V*_OE_. Genes with the regulation ratio log ≥ 2 or ≤−2 were selected.

**Figure 7:**
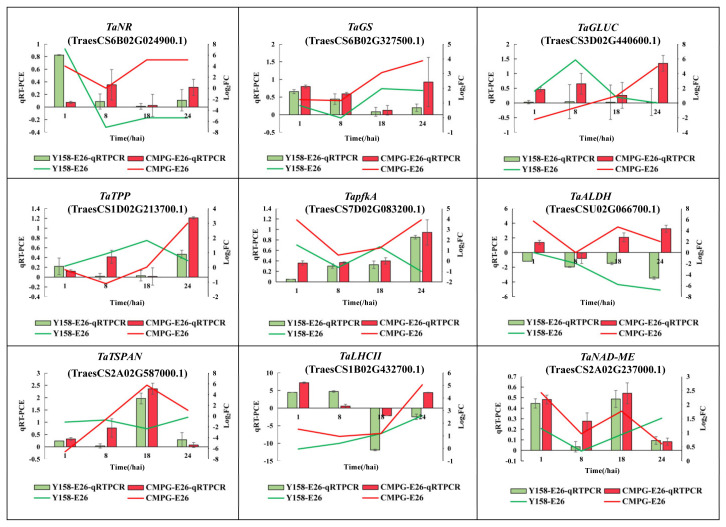
qRT-PCR and sequencing data of genes expression in *CMPG1-V*_OE_ and susceptible receptor Yangmai 158 from 1 hai to 24 hai after *Bgt* infection. The histogram represents qRT-PCR of gene expression. Green represents Yangmai 158, red represents *CMPG1-V*_OE_. Line chart represents sequencing data of genes expression. The green line represents Yangmai 158, red line represents *CMPG1-V*_OE_.

Nitrogen assimilation genes *nitrate reductase* (*NR*) and *glutamine synthetase* (*GS*) were induced apparently in *CMPG1-V*_OE_ (Figure 6a). The transcript levels of *NR* and *GS* increased more than fivefold and threefold in *CMPG1-V*_OE_, respectively. Expression levels of *NR* and *GS* increased to the maximum at 24 hai in *CMPG1-V*_OE_, which were more than those in Yangmai 158 (Figure 7). *Glutamate dehydrogenase 1* (*GDH1*) transcripts rose up to more than sixfold in *CMPG1-V*_OE_ compared with Yangmai 158. The starch and sucrose metabolism was enriched from 18 hai to 24 hai, and the transcripts of *galacturonosyltransferase* (*GAUT*) increased fivefold in *CMPG1-V*_OE_. This consisted of the *pectinesterase*, *UDP-glucuronate 4-epimerase* (*GAE*), *UDP-glucose 6-dehydrogenase* (*UGDH*), *beta-glucosidase* (*GLUC*), and *trehalose 6-phosphatephosphatase* (*TPP*), whose transcripts increased fourfold and reached their peaks at 24 hai (Figure 6b). As shown in Figure 7, expression levels of *GLUC* and *TPP* rose to the top at 24 hai in *CMPG1-V*_OE_, which were more than those in Yangmai 158 (Figure 7).

CMPG1-V activated the glycolysis/gluconeogenesis pathway. With the infection going on, the transcription levels of *ATP-dependent 6-phosphofructokinase* (*pfkA*), *pyruvate kinase* (*PK*), *pyruvate dehydrogenase E1 alpha subunit* (*PDHA*), and *aldehyde dehydrogenase* (*ALDH*) increased prominently in *CMPG1-V*_OE_ (Figure 6c). The transcripts of *PfkA* increased fourfold in *CMPG1-V*_OE_, and those of *PK* and *PDHA* showed a similar pattern. The *aldehyde dehydrogenase* transcripts were fourfold more from 18 hai until 24 hai. The expression level of *pfkA* peaked at 24 hai in *CMPG1-V*_OE_, while *ALDH* maximized in *CMPG1-V*_OE_ at 18 hai, not in Yangmai 158 (Figure 7). In addition, transcription of *NAD-dependent malic enzyme* (*NAD-ME*) and *tetraspanin-19* (*TSPAN*) associated with photosynthesis were activated. Their expression levels were more than sixfold in *CMPG1-V*_OE_ compared with those in Yangmai 158 (Figure 6d). The *chlorophyll a-b binding protein* (*LHCII*) increased sevenfold in *CMPG1-V*_OE_. The expression level of *TSPAN* maximized at 18 hai in *CMPG1-V*_OE_, *LHCII* increased to a maximum in *CMPG1-V*_OE_ at 24 hai, not in Yangmai 158. The *NAD-ME* had a significant increase in *CMPG1-V*_OE_ from 1 hai to 8 hai, which was more than that in Yangmai 158 (Figure 7). Similar results were observed in *CMPG1-V*_OE_ or Yangmai 158 tested with E31 for the induction or inactivation of nitrogen and glycolysis/gluconeogenesis metabolism (Figure S8).

### 2.6. Weighted Gene Co-Expression Network (WGCNA) of Pm Resistance Regulated by CMPG1-V

To identify crucial regulatory factors or pathways in the *CMPG1-V* defense network, the co-expression data were analyzed from all DEGs in *CMPG1-V*_OE_ and Yangmai 158 through WGCNA. The network contained 16,662 DEGs after filtering by the coefficient of variation. As shown in Figure S9, 28 module clusterings were set up and the samples collected at the four infectious time points were corresponded to each module (Figure S9a,b).

**Figure 8:**
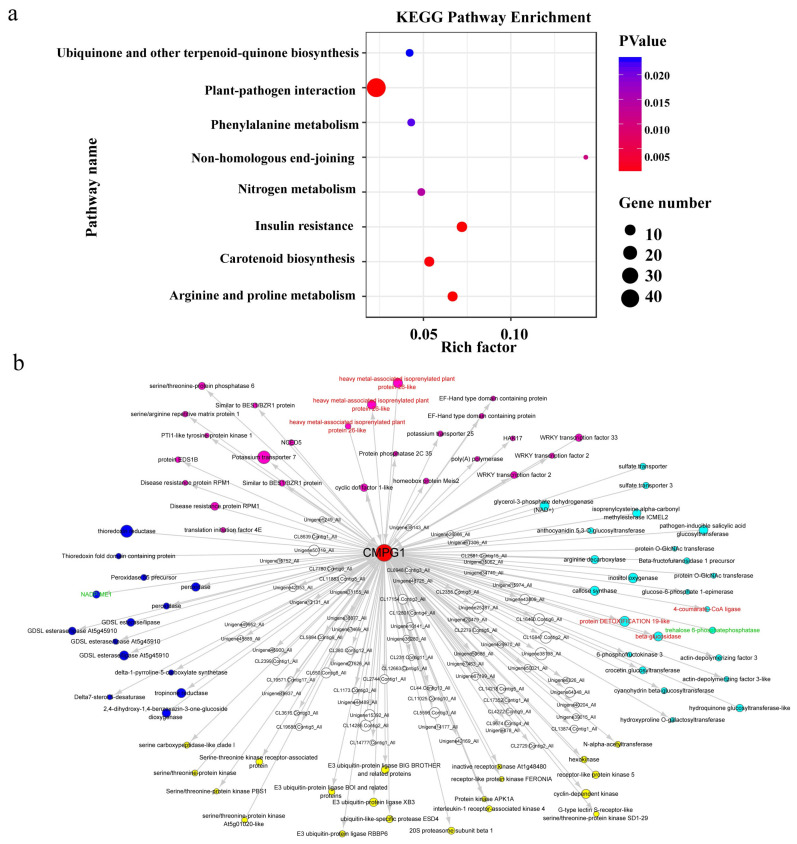
Coregulated genes expression network of *CMPG1-V* module. (**a**) KEGG enrichment analysis with all the genes in the pink module; (**b**) upstream and downstream network of *CMPG1-V*. Rose red nodes are related to signaling, light blue nodes are related to metabolism, blue nodes are related to oxidation–reduction, yellow nodes are related to protein modification. Red label font represents genes interacting with *CMPG1-V* obtained by yeast two-hybrid. Green label font represents genes having qRT-PCR results in previous study. Genes with the edge weight higher than 0.11 are visualized by Cytoscape.

In total, 2259 DEGs enriched in all modules and *CMPG1-V* were classified into the pink module (Figure S9c). KEGG enrichment analysis revealed DEGs in the pink module were closely related to plant–pathogen interaction, nitrogen metabolism, phenylalanine metabolism, and ubiquinone and terpenoid-quinone biosynthesis (Figure 8a). Twenty-six DEGs shown as rose red nodes could be categorized into plant–pathogen interaction, including *NCED5* and *PP2C35* in the ABA pathway, *EDS1B* in the SA pathway, and *RPM1* and *PTI1-like tyrosine-protein kinase 1* in disease resistance (Figure 8b). Twenty-four DEGs shown as light blue nodes were associated with phytohormone metabolism, including *beta-glucosidase* (*GLUC*), *6-phosphofructokinase 3* (*PFK3*), and *trehalose 6-phosphatephosphatase* (*TPP*) of starch and sucrose metabolism, *4-coumarate-CoA ligase* (*4CL*) of lignin metabolism, and *anthocyanidin 5,3-O-glucosyltransferase* (*GT*) of anthocyanin biosynthetic pathway (Figure 8b). In addition, 20 yellow nodes were associated with protein modification and 14 blue nodes involved in oxidation–reduction. Definitively, *CMPG1-V* interacting proteins previously identified by yeast two-hybrid assay were also identified, including 4-coumarate-CoA ligase-like, beta-glucosidase, protein DETOXIFICATION 19-like, and heavy metal-associated isoprenylated plant protein (Table S2). The DEGs including *beta-glucosidase*, *trehalose 6-phosphatephosphatase*, and *NAD-ME* were confirmed by qRT-PCR (Figure 7). Taken together, Table 1 lists the probable DEGs participating in the *CMPG1-V* defense network including protein phosphorylation modification, flavonoid metabolism, ABA/SA signaling, and oxidation homeostasis.

### 2.7. The Chromosomal Distribution of Candidate Genes

The chromosome distributions of the DEGs related to plant hormone pathways, energy metabolic signaling pathways, and in the network of the *CMPG1-V* module were surveyed through in silico mapping using Chinese Spring genomic sequences (http://www.wheatgenome.org/) as reference (Figure S10 and Table S3). In addition, all of them have homologous genes in Arabidopsis, with a similarity reached up to 80% (Table S3). As is shown in Figure S10, the 22 DEGs in plant hormone pathways were assigned to 11 different chromosomes; 29 DEGs in energy metabolism were assigned to 14 different chromosomes; and the remaining 23 DEGs in the network of *CMPG1-V* were assigned to 16 different chromosomes. Chromosome distribution analysis of the DEGs might facilitate the association study between these DEGs and previously reported genes in resistance to powdery mildew.

## 3. Discussion

Wheat powdery mildew is one of the most destructive diseases of wheat, causing extensive yield losses worldwide [17]. Due to the host or race specificity of the wheat–*Bgt* interaction, a new virulent race could rapidly evolve and emerge. Here, *CMPG1-V*, an E3 ligase gene from *H*. *villosa*, showed BSR to powdery mildew when overexpressed in wheat [18], thus the transgenic line *CMPG1-V*_OE_ and its receptor variety Yangmai 158 provide an ideal system for studying BSR during wheat–*Bgt* interaction. We identified 3542 DEGs, which were only specifically upregulated in *CMPG1-V*_OE_ after *Bgt* infection.

### 3.1. Phytohormone SA and ABA Play Important Roles in CMPG1-V-Associated Powdery Mildew Resistance

Plants are continuously exposed to diverse phytopathogenic microorganisms and have elaborated a variety of defense mechanisms to successfully avoid infection by limiting pathogen invasion and multiplication [35,36]. Plant hormone SA plays an important role in the interaction between plants and pathogens [37]. The exogenous application of SA conferred resistance against *Magnaporthe oryzae* [38]. SA-deficient NahG tobacco was hypersusceptible to powdery mildew (*Euoidium longipes*), as judged by significantly more severe powdery mildew symptoms and enhanced pathogen accumulation [39]. In a previous study, exogenous SA could increase the expression level of *CMPG1-V*, and *TaPR1* and *TaPR2* were upregulated significantly in *CMPG1-V*_OE_ when infected by *Bgt* [18]. RNA-seq showed obvious transcription level increase of the SA synthesis genes *SARD1* and *UGT* in *CMPG1-V*_OE_, hinting at the important role of the SA pathway in Pm resistance mediated by *CMPG1-V*. Arabidopsis *AtSARD1* could enhance the resistance against *Verticillium dahliae* [40]. Overexpressing *TaUGT3* enhanced fusarium head blight resistance of wheat [41]. What’s more, *TGA* TFs family positively regulates defense responses against biotrophic and necrotrophic pathogens [42], consistent with the transcription level of *TGA* accelerated in *CMPG1-V*_OE_. Thus, it will be important to elucidate the synthesis of SA during *CMPG1-V* regulating *Bgt* resistance. And what is more, the strategies targeting TAG TFs are more crucial for enhancing powdery mildew resistance during this process.

ABA, originally described for their function in response to abiotic stresses, has emerged as a crucial player in plant–pathogen interactions [43,44]. Exogenous ABA quickly induced *CMPG1-V*, suggesting that *CMPG1-V* may be involved in ABA-dependent defense response [18]. Here, we found transcripts of *SnRK2* were upregulated in all infectious periods. In Arabidopsis, SnRK2.8 phosphorylated NPR1, enhancing systemic acquired resistance (SAR) [45]. OsSAPKs (SnRK2) play positive roles in response to pathogen infection in rice [46]. ABA-inducible SnRK2-type kinase OsSAPK10 phosphorylated WRKY72, released its suppression on *AOS1*, and enhanced resistance to *Xanthomonas oryzae* pv. *oryzae* in rice [47]. Besides, PP2C, a negative regulator in ABA signaling, was significantly downregulated in *CMPG1-V*_OE_ after *Bgt* inoculation compared with Yangmai 158. Some rice PP2Cs subgroup K negatively regulate *X. oryzae* pv. *oryzae* infection [48]. Thus, PP2Cs are thought to play a specific role in *CMPG1-V*-associated defense pathways, and ABA-dependent defense signaling possibly plays a positively regulatory role in *CMPG1-V* resistance network.

Chromosome distribution analysis of the DEGs provided information to identify whether these DEGs were associated with previously identified genes or QTL. By gene expression comparison between resistant and susceptible durum wheat lines, a *WAK2* gene in the mapped region of *QFhb.mgb-2A* was identified and responsible for Fusarium Head Blight resistance [49,50]. The DEGs which might be involved in *CMPG1-V* regulatory network have been mapped in silico onto chromosome regions of wheat (Table S3). We have found that a CIPK29 gene (Unigene49646_All) which was located in the terminal region 2BS might be associated with powdery-mildew-resistant gene *Ml5323* in the interval of 2BS FL0.84-1.00 [49]. CIPKs were reported to play roles in the ABA signaling pathway [51]. This finding might provide clues to the cloning and elucidation of the resistant mechanism mediated by *Ml5323*.

### 3.2. CMPG1-V Reprogrammed Starch and Sucrose Metabolism/Photosynthesis in Response to Bgt Infection

When a plant defends against pathogen invasion, levels of resistance in the whole plant are influenced by systemic signals mediated by plant hormones [52]. Plant hormones can coordinate various signal pathways to mediate metabolism under pathogen infection [1]. NR-mediated NO generation plays a key role in protecting plants from abiotic stresses through activating antioxidant enzymes [53]. GS1 can be induced during development of the infectious process [54]. We observed that, in response to *Bgt* infection, the nitrogen assimilation genes *NR* and *GS* were upregulated apparently in *CMPG1-V*_OE_. What is more, typical stress metabolite trehalose plays an important role in regulation of defense response against pathogens. Some selected *SlTPPs* could be inducted by *Botrytis cinerea* and *Pseudomonas syringae* pv. *tomato* (*Pst*) DC3000 as well as defense signaling hormones [55]. We found *TPP* involving starch and sucrose metabolism and *pfkA*, *PK*, *PDHA* involved in glycolysis/gluconeogenesis were induced notably in *CMPG1-V*_OE_. The *GAPDHs*, multifunctional enzymes in glycolysis, have been proven to be involved in regulation of ROS, autophagy, and plant immune responses [56]. Photosynthesis also modulates plant defense responses induced by pathogen infection and abiotic signals such as light, circadian rhythm, and temperature [57]. During infection with *Colletotrichum higginsianum*, loss-of-function mutants of *NADP-ME2 (nadp-me2*) showed enhanced susceptibility [58]. The transcription levels of *NAD-dependent malic enzyme*, *chlorophyll a-b binding protein*, and *tetraspanin-19* were upregulated remarkably in *CMPG1-V*_OE_, compared to those in Yangmai 158. Thus, the reprogramming of starch/sucrose metabolism and photosynthesis pathways played an important role in the *CMPG1-V* defense response to *Bgt* infection.

### 3.3. CMPG1-V Exerts Its Distinct Defense Response by Reprogramming a Specific Network

Plants have evolved innate immune systems that recognize the presence of potential pathogens and initiate effective defense responses. E3 ubiquitin ligase plays a crucial role in the plant immunity process [2]. There are, however, a few notable highlights or differences in resistance mechanisms against *Bgt* in *CMPG1-V*. WGCNA of *CMPG1-V* module revealed its close relation with phosphorylation modification, flavonoid metabolism, oxidation homeostasis, and WRKY. Crosstalk between ubiquitination and phosphorylation modification plays a key role in controlling the defense signaling events to ensure plant pathogen resistance [2]. Crosstalk between posttranslational modifications, such as ubiquitination and phosphorylation, play key roles in controlling the duration and intensity of signaling events to ensure cellular homeostasis [59]. Phosphorylation events contribute additively to the stabilization of PUB22 in response to the perception of pathogen-associated molecular patterns (PAMPs) [59]. Phosphorylation of NPR1 could switch autoubiquitination activity of BTB^NPR1^ to substrate ubiquitination by enhancing its affinity to targets such as TGA2 [60]. For oxidation homeostasis, E3 ubiquitin ligase can induce ROS to enhance the resistance level. Knockdown of stress inducible *OsSRFP1*, encoding an E3 ubiquitin ligase with transcriptional activation activity, conferred abiotic stress tolerance through enhancing antioxidant protection in rice [61]. Arabidopsis E3 ubiquitin ligase PUB13 regulated chitin receptor LYSIN MOTIF RECEPTOR KINASE5 and induced rapid responses, such as the production of ROS [62]. Moreover, other E3 ligases can interact with WRKY to enhance the resistance. The E3 ligase likely to be involved in plant immunity is UPL5, which was first identified by a yeast two-hybrid screen. UPL5 interacted with WRKY53, a transcription factor acting positively in leaf senescence [63]. The Chinese wild grapevine (*Vitis pseudoreticulata*) E3 ligase EIRP1 activated plant defense responses by inducing proteolysis of the VpWRKY11 transcription factor [64]. However, there are few studies about ubiquitination in plant immunity related with flavonoid metabolism. In this study, we found flavonoid metabolism may have an influence on ubiquitination in plant immunity. This will give us a new perspective to clarify the resistance function of *CMPG1-V* in wheat powdery mildew resistance. On all accounts, these candidates will provide extensive insights into molecular mechanisms of *CMPG1-V*-associated broad-spectrum powdery mildew resistance in wheat.

## 4. Materials and Methods

### 4.1. Plant Material and Fungal Isolates

Yangmai 158 is a moderate powdery-mildew-susceptible wheat variety and it is susceptible to Bgt isolates E26 or E31. Overexpression of CMPG1-V in Yangmai 158 enhanced its broad-spectrum resistance. Bgt isolates E26 and E31 were collected from Institute of Plant Protection, Chinese Academy of Agricultural Sciences, Beijing, China. They are maintained on seedlings of susceptible wheat variety Sumai 3 in a spore-proof greenhouse. All seedlings were grown in a growth chamber with 20 °C/16 °C (day/night), 16 h/8 h (light/dark). CMPG1-V transgenic line (CMPG1-V_OE_) and the receptor variety Yangmai 158 were inoculated with Bgt isolates E26 and E31 at the two-leaf stage and RNAs were isolated at 1 h, 8 h, 18 h, and 24 h before and after Bgt inoculation, followed by freezing in liquid nitrogen for subsequent RNA extraction.

### 4.2. RNA-Seq Library Construction and Sequencing

Total RNA extraction was performed using the Trizol reagent (Invitrogen, Waltham, MA, USA), according to the manufacturer’s instructions. RNA concentration was measured using a NanoDrop spectrophotometer and 1.2% agarose gel electrophoresis.

Sample detection and sequencing were performed by BGI-Shenzhen, Shenzhen Beijing. After extracting total RNA and treating with DNase I, Oligo(dT) was used to isolate mRNA. Mixed with the fragmentation buffer, the mRNA were fragmented. Then, cDNA was synthesized using the mRNA fragments as templates. Short fragments were purified and resolved with EB buffer for end reparation and single nucleotide A (adenine) addition. After that, the short fragments were connected with adapters. The suitable fragments were selected for the PCR amplification. During the QC steps, Agilent 2100 Bioanaylzer and ABI Step One Plus Real-Time PCR System were used in quantification and qualification of the sample library. Then, the library was sequenced using Illumina HiSeq 4000 (Illumina, San Diego, CA, USA). The raw data and processed data could be obtained from the NCBI website address. The initial short reads data sets were available at the NCBI Short Read Archive (SRA) with the accession number SRR12416499-SRR12416522.

### 4.3. Sequencing Reads Filtering and De Novo Assembly

To obtain clean reads, raw reads, including reads with adaptors, reads in which unknown bases represented more than 5% of the total bases, and low-quality reads (percentage of low-quality bases with a quality value ≤ 10 in more than 20% of a read), were removed. We used Trinity to perform de novo assembly with clean reads that PCR duplication removed (in order to improve the efficiency), then used Tgicl to cluster transcripts to Unigenes. Trinity: version: v2.0.6, parameters: --min_contig_length 150 --CPU 8 --min_kmer_cov 3 --min_glue 3 --bfly_opts ‘-V 5 --edge-thr=0.1 --stderr’. Tgicl: version: v2.0.6, parameters: -l 40 -c 10 -v 25 -O ‘-repeat_stringency 0.95 -minmatch 35 -minscore 35′.

### 4.4. Unigene Functional Annotation and Expression Calculation

NT (ftp://ftp.ncbi.nlm.nih.gov/blast/db), NR (ftp://ftp.ncbi.nlm.nih.gov/blast/db), GO (http://geneontology.org), COG (http://www.ncbi.nlm.nih.gov/COG), KEGG (http://www.genome.jp/kegg), Swiss Prot (http://ftp.ebi.ac.uk/pub/databases/swissprot), and Inter Pro (http://www.ebi.ac.uk/interpro) are functional databases. We used Blast [65] align Unigenes to NT, NR, COG, KEGG, and Swiss Prot to get the annotation, used Blast2GO [66] with NR annotation to get the GO annotation, and used InterProScan5 [67] to get the Inter Pro annotation. We mapped clean reads to Unigenes using Bowtie2 [68], and then calculated gene expression level with RSEM. Blast: version: v2.2.23, parameters: default, website: http://blast.ncbi.nlm.nih.gov/Blast.cgi. Blast2GO: version: v2.5.0, parameters: default, website: https://www.blast2go.com. InterProScan5: version: v5.11-51.0, parameters: default, website: https://code.google.com/p/interproscan/wiki/Introduction. Bowtie2: version: v2.2.5, parameters: -q --phred64 --sensitive --dpad 0 --gbar 99999999 --mp 1,1 --np 1 --score-min L,0,-0.1 -I 1 -X 1000 --no-mixed --no-discordant -p 1 -k 200, website: http://bowtie-bio.sourceforge.net/ Bowtie2 /index.shtml. RSEM: version: v1.2.12, parameters: default, website: http://deweylab.biostat.wisc.edu/RSEM.

### 4.5. Detection and Analysis of Differentially Expressed Gene

We detected DEGs with PossionDis as requested and parameters were set as Fold Change ≥ 2.00 and FDR ≤ 0.001. With the GO annotation result, we classified DEGs according to official classification, and we also performed GO functional enrichment using phyper, a function of R (version: v3.4.2). With the KEGG annotation result, we classified DEGs according to official classification, and we also performed pathway functional enrichment using phyper, a function of R. We calculated false discovery rate (FDR) for each p-value; in general, the terms with FDR not larger than 0.001 were defined as significantly enriched. Hierarchical clustering and heat map generation were performed in R. The gene expression data were log2-transformed and then quantile-normalized prior to generating the heat map for direct comparison of the data.

### 4.6. Weighted Gene Co-Expression Network Analysis and Visualization

Co-expression networks were constructed using the WGCNA package in R. We used the log2-transformed FPKM values, and replaced values smaller than one by zero. In total, 16,662 genes were used for the WGCNA analysis. The modules were obtained using the automatic network construction function block wise Modules with default settings. Parameters were set as follows: the power was 20, TOM-Type was unsigned, min Module Size was 30, and merge Cut Height was 0.25. We inferred an undirected, weighted network. Within the network, clusters of genes with similar expression patterns, called modules, were then inferred using a clustering of the Topological Overlay Matrix. The eigengene value was calculated for each module and 16,662 genes were clustered into 27 specific modules. The networks were visualized using Cytoscape _v.3.5.1.

### 4.7. Quantitative Real-Time Polymerase Chain Reaction (qRT-PCR) Assay

All selected genes were used for SYBR green real-time RT-PCR; primers are listed in Table S4. The Tubulin gene was used as the internal control gene. The qRT-PCR reaction for target gene transcript amplification was carried out in a final volume of 20 μL containing 20 ng cDNA, 0.2 μM of each primer, 2× AceQ qPCR SYBR Green Master Mix (Vazyme, Nanjing, China). The PCR reaction conditions were denaturation at 95 °C for 5 min followed by 40 cycles of 95 °C for 10 s, annealing at the appropriate temperature (from 57 to 61 °C) for 30 s, extension at 72 °C for 30 s, followed by 95 °C for 15 s, 60 °C for 1 min, then 95 °C for 15 s to obtain melt curves to ensure primer specificity. All reactions were done in triplicate. Reactions were performed on a Bio-Rad IQ single-color Real-Time PCR detection System (Bio-Rad, Hercules, CA, USA). The data were compiled from the mean Ct values of all the replicates after normalizing with the Ct values of the endogenous control. The relative expression level in terms of fold change was calculated using the 2^−^^ΔΔCT^ method [69]. All the qRT-PCRs had three biological replicates.

### 4.8. Statistical Analysis

All statistical analyses were performed using SPSS version 13.0, and one-way ANOVA was performed with a homogeneity of variance test, followed by an LSD test to check for quantitative differences between treatments. *p* < 0.05 was set as the significance cut-off.

## 5. Conclusions

**Figure 9:**
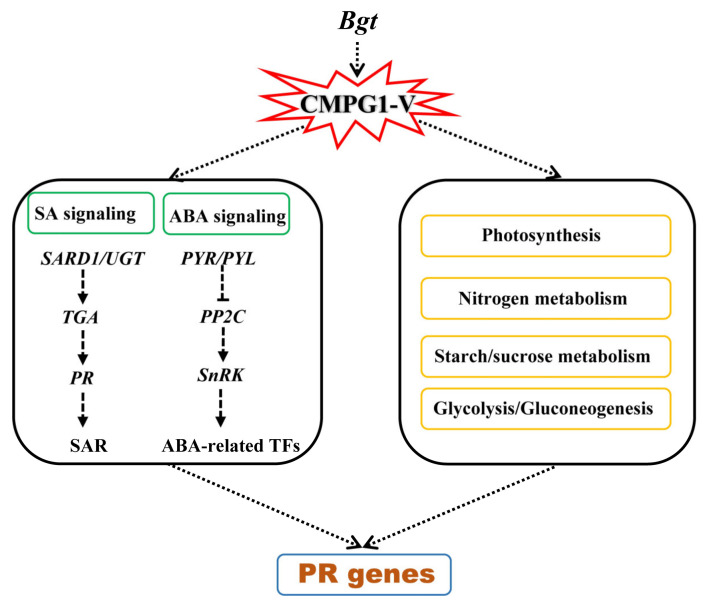
Proposed model for the molecular mechanism of powdery mildew resistance mediated by *CMPG1-V*.

In this study, we analyzed the temporal specificity of Pm resistance regulated by *CMPG1-V* at the whole genome level, represented by four time points after *Bgt* infection. We suspected that when the pathogen attacked, the plant activated *CMPG1-V*, then enhanced hormone defense signaling through SA and ABA signaling. Activation of C/N and photosynthesis metabolism provided energy during wheat–*Bgt* interaction, which affected the expression of downstream PR genes (Figure 9). Moreover, phosphorylation modification probably regulated different transcription of genes in defense signaling and related metabolisms. Our findings point to strengthening the utility of *CMPG1-V* in enhancing BSR to powdery mildew.

## Figures and Tables

**Table 1:** Different expressed genes related with *CMPG1-V* Type.

	Gene Annotation	Triticum Aestivum ID (Chinese Spring)	Ortholog ID (Arabidopsis)	Function
protein phosphorylation	receptor-like protein kinase 5	TraesCS1D02G344700.1	AT5G25930.1	membrane signal transduction [20]
	serine/threonine- kinase receptor	TraesCS5A02G189200.1	AT3G15610.1	plant developmental [21]
	hexokinase	TraesCS3D02G276200.1	AT5G25930.1	membrane signal transduction [20]
	interleukin-1 receptor-associated kinase	TraesCS4D02G286100.1	AT2G02800.1	early elicitor signaling [22]
	serine/threonine-protein kinase	TraesCS7D02G165300.1	AT2G05940.1	PTI [23]
	serine/threonine-protein kinase SD1-29	TraesCS2D02G217400.1	AT4G21390.2	early elicitor signaling [22]
	protein kinase APK1A	TraesCS7A02G163300.1	AT2G05940.1	PTI [23]
	serine/threonine-protein kinase PBS1	TraesCS2A02G348300.1	AT3G59360.1	cell wall biosynthesis [24]
	receptor-like protein kinase FERONIA	TraesCS5D02G336300.1	AT3G51550.1	male–female interaction [25]
	inactive receptor kinase	TraesCS4A02G267300.1	AT1G48480.1	pathogen infection [26]
flavonoid biosynthetic	salicylic acid glucosyltransferase	TraesCS5A02G149600.1	AT1G05675.1	SA/JA-mediated defense [27]
	hydroquinone glucosyltransferase-like	TraesCS7A02G216000.1	AT4G01070.1	cell wall lignification [28]
	crocetin glucosyltransferase	TraesCS3D02G120200.1	AT4G15550.1	seed germination [29]
	cyanohydrin beta-glucosyltransferase	TraesCS5D02G324600.1	AT1G22360.1	cell cycle regulation [30]
	anthocyanidin 5, 3-O-glucosyltransferase	TraesCS2B02G012000.1	AT3G16520.3	unknown
	protein O-GlcNAc transferase	TraesCS1A02G351900.1	AT3G18170.1	unknown
	protein O-GlcNAc transferase	TraesCS1D02G358500.1	AT3G18170.1	unknown
signaling transcription	WRKY transcription factor 2	TraesCS3A02G209800.1	AT5G26170.1	JA defense responses [31]
	WRKY transcription factor 2	TraesCS5B02G257300.1	AT5G56270.1	seed germination by ABA [32]
	WRKY transcription factor 33	TraesCS1D02G292700.1	AT2G38470.1	ABA signaling [33]
oxidative stress	peroxidase 55 precursor	TraesCS5B02G287600.1	AT5G14130.1	unknown
	peroxidase	TraesCS7B02G381400.1	AT4G39720.1	unknown
	peroxidase	TraesCS1B02G420500.1	AT3G14180.1	seedling development [34]

## Supplementary Materials

Supplementary materials can be found at https://www.mdpi.com/1422-0067/21/17/5967/s1.

## Abbreviations

4CL	4-coumarate-CoA ligase
ABA	abscisic acid
AGT	appressorial germ tube
ALDH	aldehyde dehydrogenase
Bgt	Blumeria graminis f. sp. tritici
bp	base pair
BSR	broad-spectrum resistance
DEG	differentially expressed gene
ET	ethylene
GAE	UDP-glucuronate 4-epimeras
GAUT	galacturonosyltransferase
GDH1	glutamate dehydrogenase 1
GLUC	beta-glucosidase
GO	Gene Ontology
GS	glutamine synthetase
GT	anthocyanidin 5,3-O-glucosyltransferase
HA	hyphal appressoria
JA	jasmonic acid
KEGG	Kyoto Encyclopedia of Genes and Genomes
LHCII	chlorophyll a-b binding protein
NAD-ME	NAD-dependent malic enzyme
NR	nitrate reductase
PDHA	pyruvate dehydrogenase E1 alpha subunit
PFK3	6-phosphofructokinase 3
pfkA	ATP-dependent 6-phosphofructokinase
PGT	primary germ tube
PK	pyruvate kinase
Pm	powdery mildew
PR	pathogenesis-related gene
PUB	plant U-box type E3 ubiquitin ligases
RNA-seq	RNA-sequencing
SA	salicylic acid
SARD1	SAR DEFICIENT1
SnRK	SNF1-related protein kinase
TPP	trehalose 6-phosphatephosphatase
TSPAN	tetraspanin-19
UGDH	UDP-glucose 6-dehydrogenase
UGT	UDP-glycosyltransferase
WGCNA	Weighted Gene Co-Expression Network Analysis
