# Supplementary material for: The Regulatory Network of CMPG1-V in Wheat–Blumeria graminis f. sp. tritici Interaction Revealed by Temporal Profiling Using RNA-Seq
Source: Int J Mol Sci. 2020 Aug 19;21(17):5967. doi: 10.3390/ijms21175967 (PMC7504233; doi:10.3390/ijms21175967)
Supplement: Supplementary file 1 [file ijms-21-05967-s001.zip › Supplementary/ijms-885234-Supplementary.pdf]

International Journal of
Molecular Sciences

Supplementary figures and tables

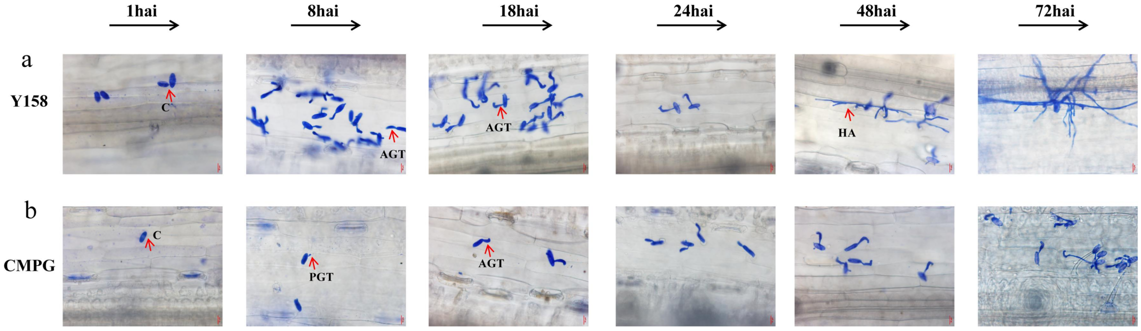

Figure S1. The development and infection cycle of *Bgt* in Yangmai 158 (a) and in *CMPG1-V*$_{OE}$ (b). C: a single conidium; PGT: primary germ tube; AGT: appressorial germ tube; HA: hyphal appressoria. Bar=10um.

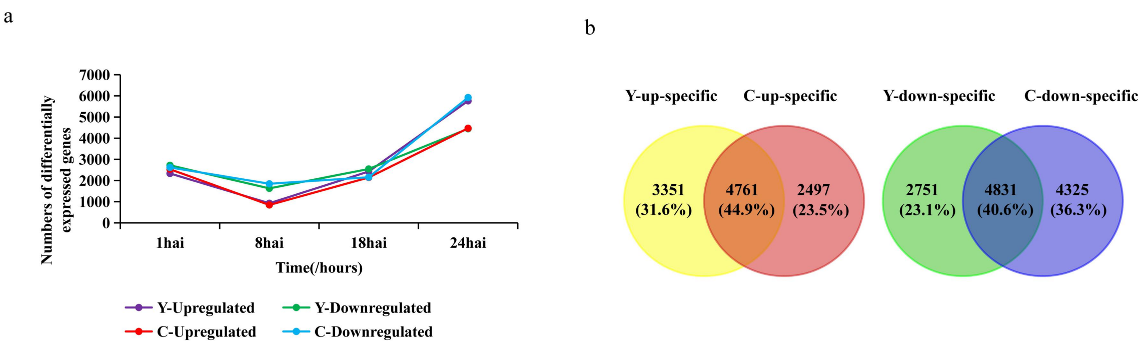

Figure S2. Differentially expressed genes (DEGs) in *CMPG1-V*$_{\mathrm{OE}}$ and susceptible receptor Yangmai 158 after E31 infection. (a) DEGs of *CMPG1-V*$_{\mathrm{OE}}$ and Yangmai 158 in different infection points. Purple and green represent upregulated and downregulated DEGs in Yangmai 158, red and blue represent upregulated and downregulated DEGs in *CMPG1-V*$_{\mathrm{OE}}$. (b) Venn diagram of upregulated and downregulated DEGs in *CMPG1-V*$_{\mathrm{OE}}$ and Yangmai 158. (FDR <= 0.001; genes with the regulation ratio log $\geq$ 2 or $\leq$ -2 were selected).

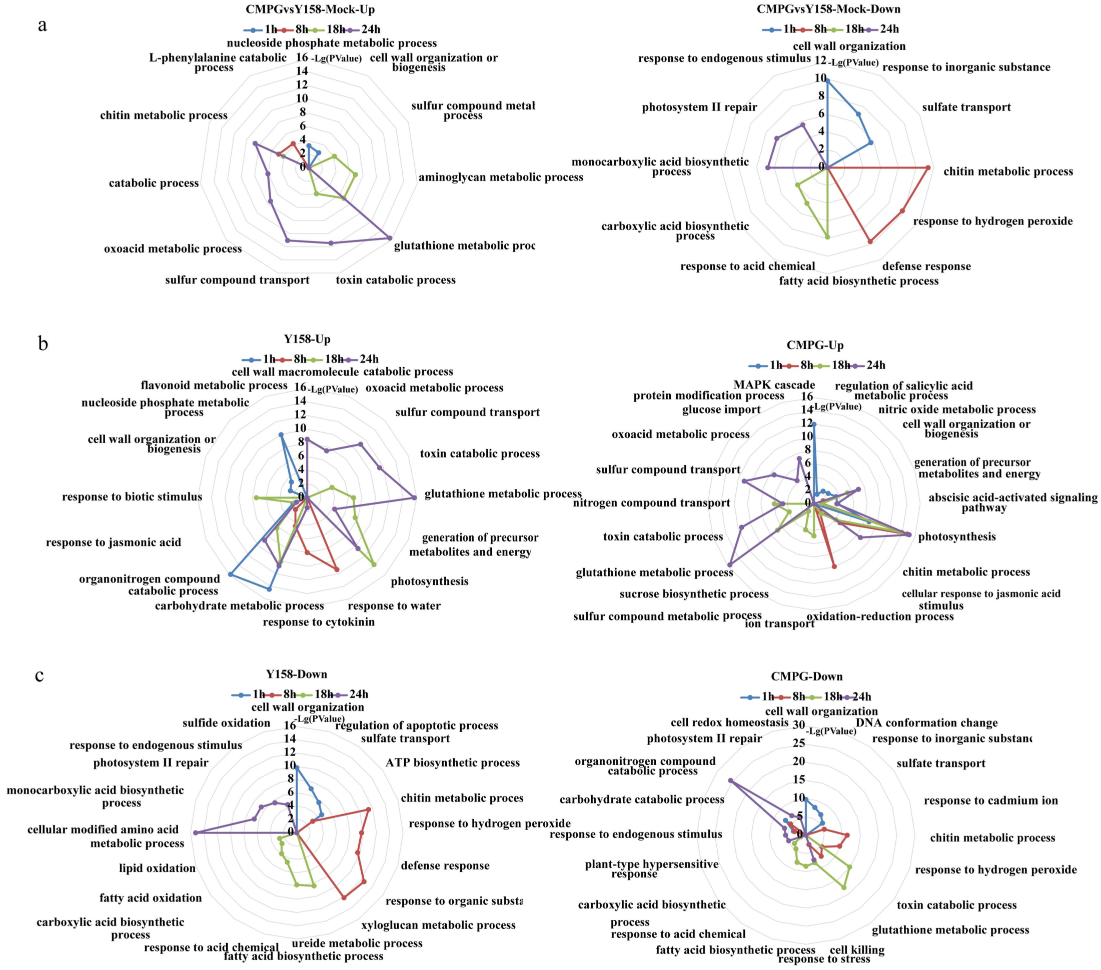

Figure S3. GO classification enrichment in *CMPG1-V*$_{OE}$ and susceptible receptor Yangmai 158 before and after E26 infection. (a) GO analysis in *CMPG1-V*$_{OE}$ and Yangmai 158 from upregulated and downregulated DEGs from 1 hai to 24 hai before *Bgt* infection. (b) GO analysis in Yangmai 158 and *CMPG1-V*$_{OE}$ from 1 hai to 24 hai from common upregulated genes after *Bgt* infection. (c) GO analysis in Yangmai 158 and *CMPG1-V*$_{OE}$ from 1 hai to 24 hai from common downregulated genes after *Bgt* infection. All DEGs were selected as follows: *P* value < 0.05; genes with the regulation ratio log $\geq$ 2 or $\leq$ -2 were selected.

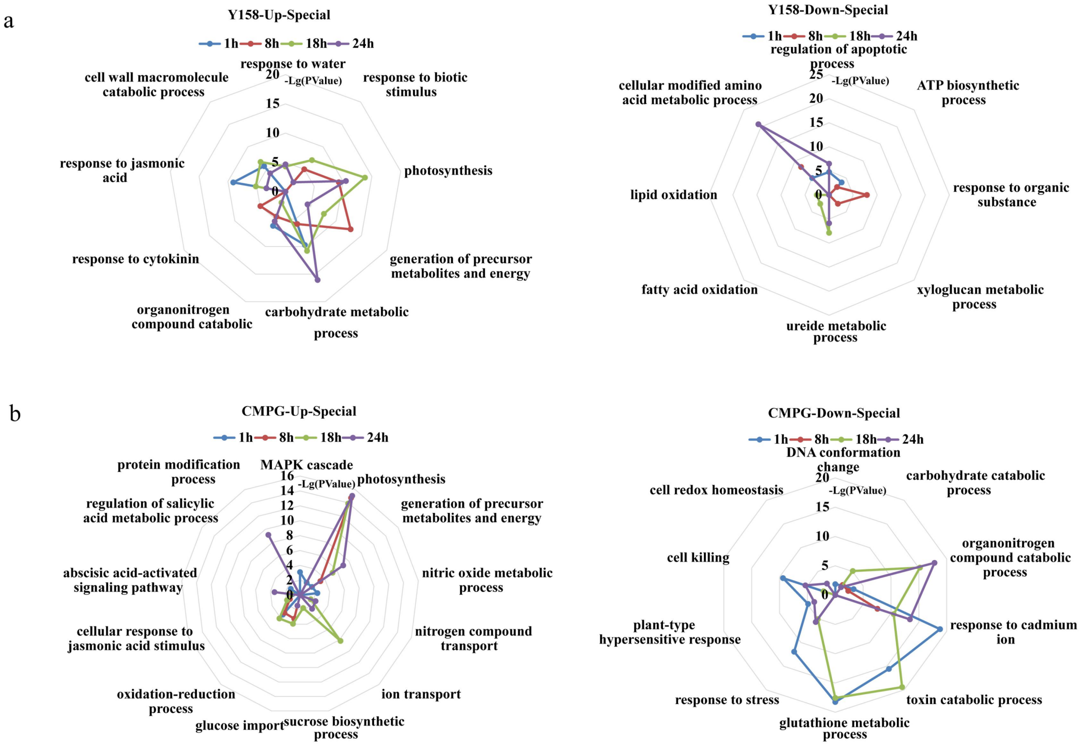

Figure S4. Specificity categaries in susceptible receptor Yangmai 158 (a) and *CMPG1-V*$_{OE}$ (b) after E31 infection. Upregulated and downregulated DEGs from 1 hai to 24 hai were selected as follows: *P* value < 0.05; genes with the regulation ratio log ≥ 2 or ≤ -2 were selected.

a

b

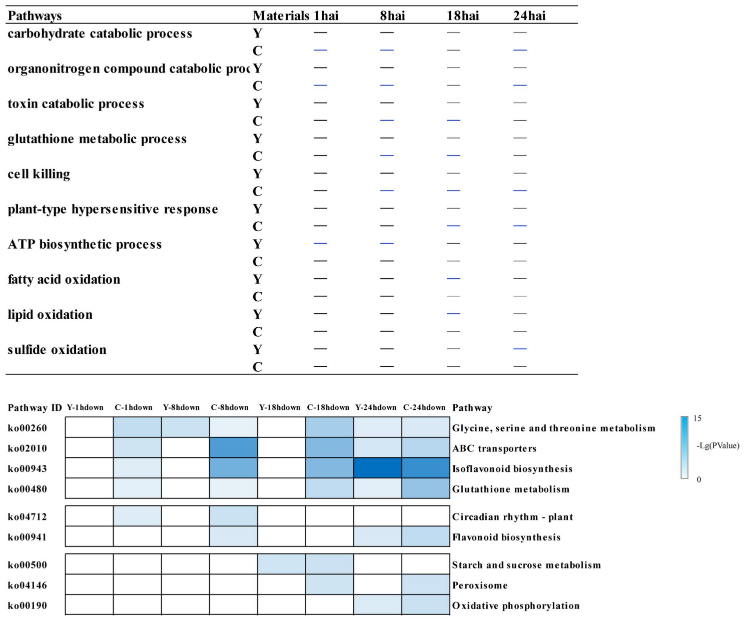

Figure S5. GO classification and KEGG pathways enrichment in *CMPG1-V*$_{OE}$ and susceptible receptor Yangmai 158 from 1 hai to 24 hai after E26 infection. (a) GO analysis in Yangmai 158 and *CMPG1-V*$_{OE}$ from1 hai to 24 hai from special downregulated genes. (b) Heat map showing the *P* value significance of enriched KEGG pathways of downregulated DEGs in *CMPG1-V*$_{OE}$ and Yangmai 158 from 1 hai to 24 hai. Y represents Yangmai 158, C represents *CMPG1-V*$_{OE}$. The colors white and blue represent low and high expression levels. Downregulated pathways were selected as follows: *P* value < 0.05; genes with the regulation ratio log ≥ 2 or ≤ -2 were selected.

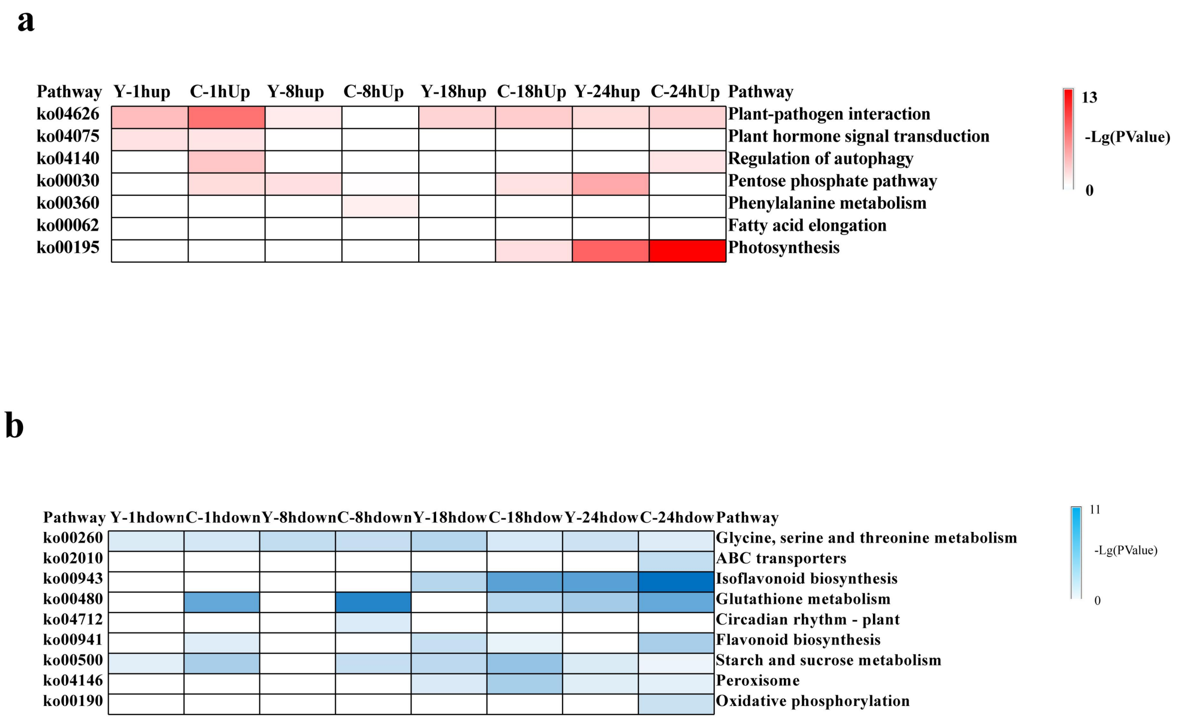

Figure S6. KEGG pathways enrichment in *CMPG1-V*$_{OE}$ and susceptible receptor Yangmai 158 from 1 hai to 24 hai after E31 infection. (a) Heat map showing the *P* value significance of enriched KEGG pathways of upregulated DEGs in *CMPG1-V*$_{OE}$ and Yangmai 158 from 1 hai to 24 hai. The colors white and red represent low and high expression levels. (b) Heat map showing the *P* value significance of enriched KEGG pathways of downregulated DEGs in *CMPG1-V*$_{OE}$ and Yangmai 158 from 1 hai to 24 hai. Y represents Yangmai 158, C represents *CMPG1-V*$_{OE}$. The colors white and blue represent low and high expression levels. Downregulated pathways were selected as follows: *P* value < 0.05; genes with the regulation ratio log ≥ 2 or ≤ -2 were selected.

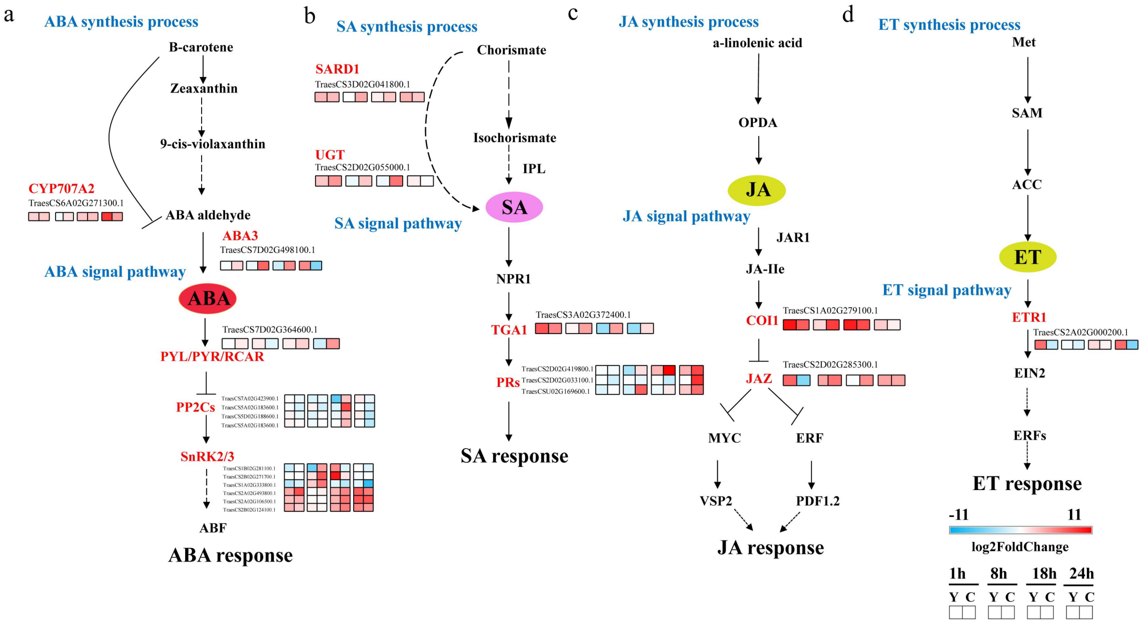

Figure S7. Differentially expressed genes related to plant hormone pathways in *CMPG1-V*$_{OE}$ and susceptible receptor Yangmai 158 from 1 hai to 24 hai after E31 infection. (a) ABA pathway; (b) SA pathway; (c) JA pathway; (d) ET pathway. Heat map showing the gene expression. The colors blue, white and red represent low, medium and high expression levels, respectively. Y represents Yangmai 158, C represents *CMPG1-V*$_{OE}$. Genes with the regulation ratio log $\geq$ 2 or $\leq$ -2 were selected.

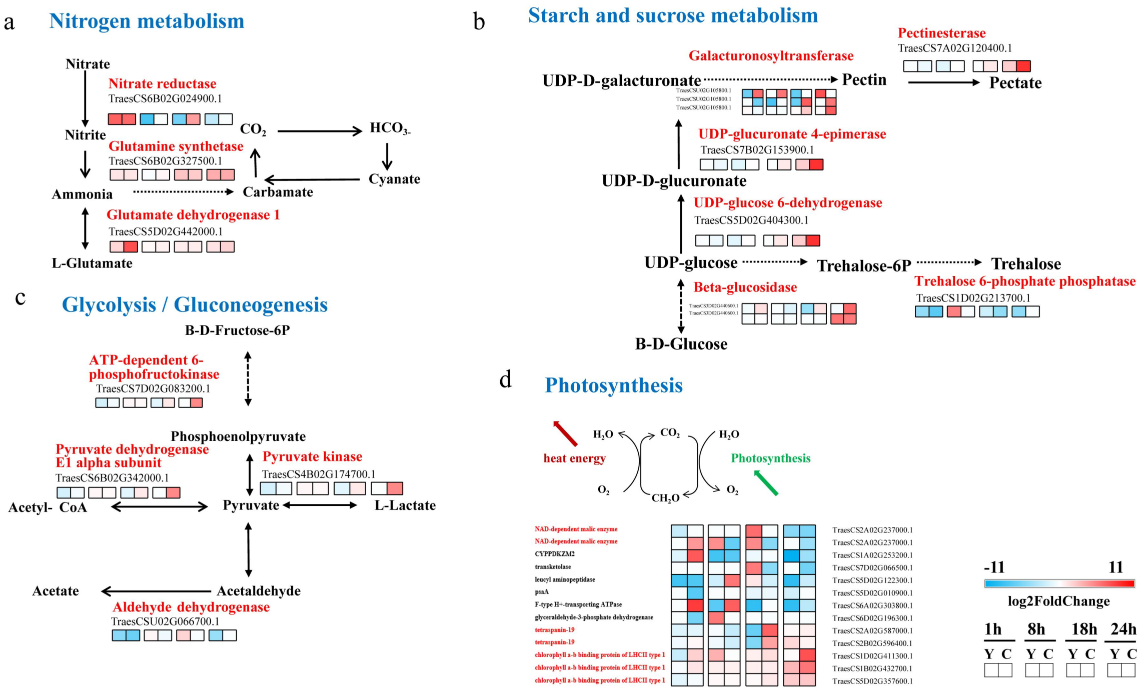

Figure S8. *CMPG1-V*$_{OE}$ and Yangmai 158 DEGs related to four energy metabolic signaling pathways after E31 infection. (a) Nitrogen metabolism; (b) starch and sucrose metabolism; (c) glycolysis / gluconeogenesis; (d) photosynthesis. Heat map showing the gene expression. The colors blue, white and red represent low, medium and high expression levels, respectively. Y represents Yangmai 158, C represents *CMPG1-V*$_{OE}$. Genes with the regulation ratio log ≥ 2 or ≤ -2 were selected.

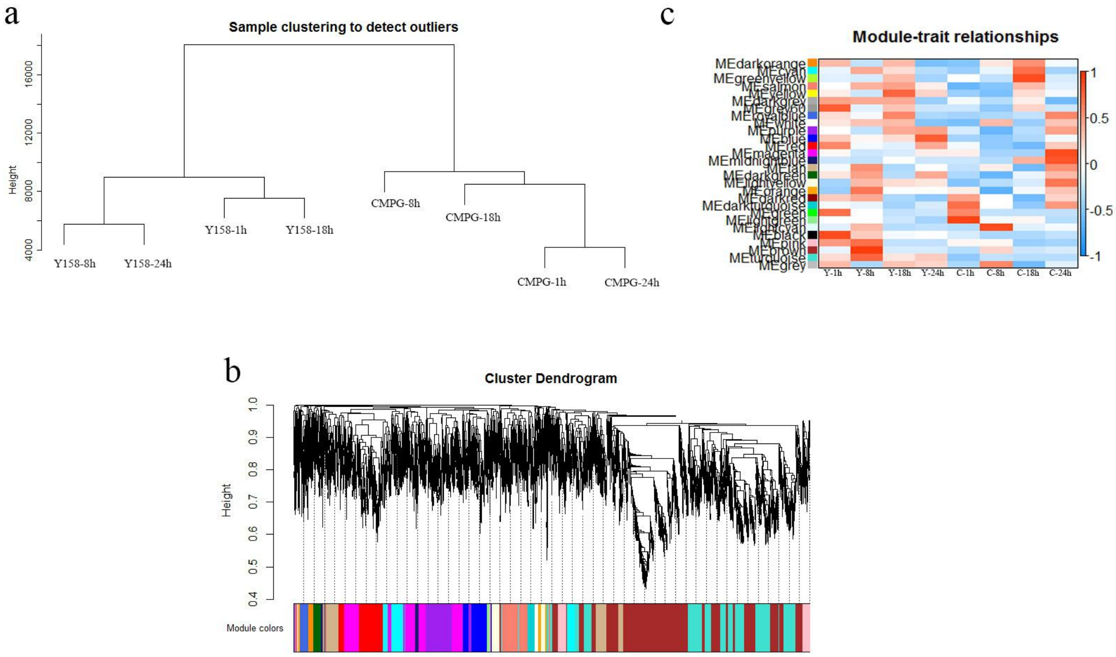

Figure S9. (a) Cluster analysis between samples; (b) Module cluster analysis; Each row corresponds to a module. (c) Module and sample correlation analysis. Hierarchical cluster tree showing co-expression modules identified by WGCNA. Each leaf in the tree is one gene.

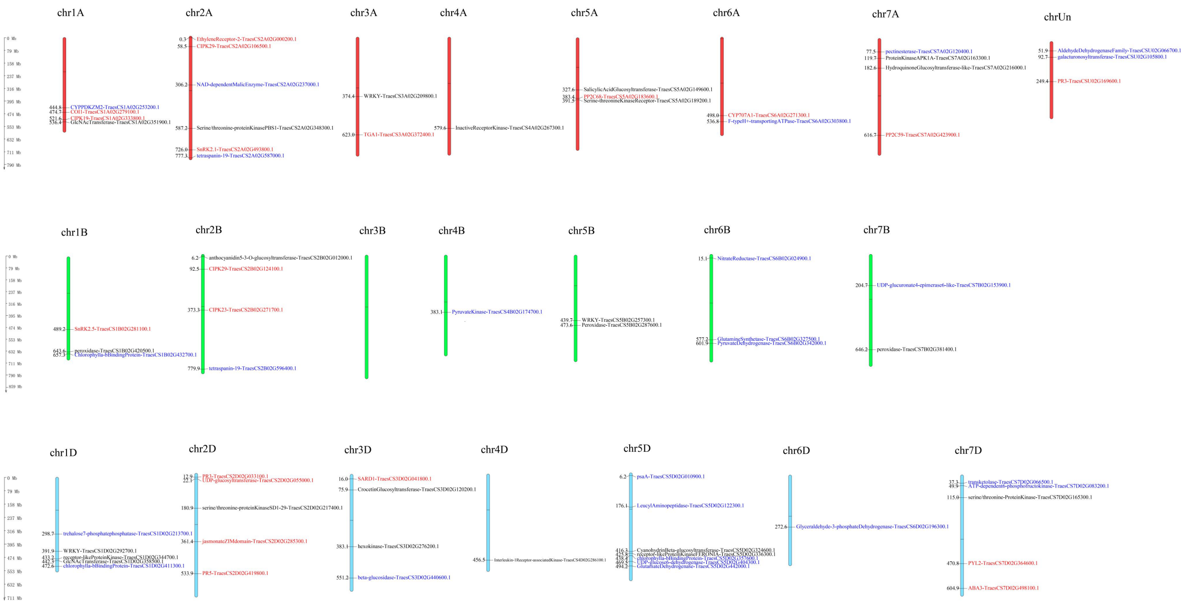

Figure S10. The chromosomal distribution of candidate genes. The name of each gene is to the right of each chromosome. Gene names labeled with red, blue and black indicate that genes related to plant hormone pathways, genes related to energy metabolic signaling pathways and genes in network of *CMPG1-V* module, respectively.

83 **Table S1.** Summary of RNA-Seq reads after filtering

Sample	CleanReads Q20（%）	CleanReads Q30（%）	CleanReads Ratio（%）	Mean Length	N50	GCcontent（%）
CMPG1-V-1h-E26	93.73	85.94	90.01	757	1195	53
CMPG1-V-1h-E31	95.3	88.95	94.8	815	1286	52.64
CMPG1-V-1h-Mock	94.49	87.33	92.36	750	1169	52.69
CMPG1-V-8h-E26	93.85	86.19	93.64	772	1214	52.39
CMPG1-V-8h-E31	94.03	86.48	93.84	759	1212	52.78
CMPG1-V-8h-Mock	93.4	85.47	92.26	790	1265	51.79
CMPG1-V-18h-E26	93.67	85.87	93.36	772	1214	52.39
CMPG1-V-18h-E31	93.66	85.81	93.79	759	1212	52.78
CMPG1-V-18h-Mock	93.56	85.71	90.87	790	1265	51.79
CMPG1-V-24h-E26	95.61	89.54	95.17	779	1233	52.59
CMPG1-V-24h-E31	94.01	86.44	93.18	746	1189	52.05
CMPG1-V-24h-Mock	95.59	89.52	91.65	769	1230	52.47
Y158-1h-E26	94.47	87.36	91.63	744	1157	52.86
Y158-1h-E31	94.76	87.81	89.87	766	1210	53.3
Y158-1h-Mock	94.65	87.64	90.14	773	1220	53.41
Y158-8h-E26	95.59	89.48	92.41	825	1303	53.57
Y158-8h-E31	94.48	87.34	91.47	803	1272	53.26
Y158-8h-Mock	93.61	85.67	90.32	779	1240	53.26
Y158-18h-E26	94.58	87.53	91.13	743	1166	52.9
Y158-18h-E31	94.56	87.47	92.35	775	1243	52.53
Y158-18h-Mock	95	88.39	93.7	818	1302	52.64
Y158-24h-E26	93.03	84.63	89.41	735	1152	53.43
Y158-24h-E31	94.96	88.31	94.14	795	1253	52.83
Y158-24h-Mock	94.69	87.75	94.79	798	1261	52.91

84

85

Table S2. Proteins interacting with CMPG1-V

**Gene annotation**	**classification**	**ID**	**Weight**
beta-glucosidase	glucan catabolic process	CL16999.Contig6_All	0.11
4-coumarate-CoA ligase	phenylpropanoid metabolic process	Unigene181_All	0.04
protein DETOXIFICATION 19-like	drug transmembrane transport	Unigene63720_All	0.13
heavy metal-associated isoprenylated plant protein 26-like	metal ion transport	Unigene67134_All	0.1

90

91 **Table S3.** Detailed list of the differentailly expressed genes

Gene	Categories	Unigene ID	Arabidopsis thaliana ID	Identities	Triticum aestivum ID	Identities	Triticum aestivum Position
CYP707A1	Hormone pathways	CL3728.Contig5_All	AT4G19230.1	71.689	TraesCS6A02G271300.1	99.376	chr6A:498006802-498009548
abscisic aldehyde oxidase 3(AAO3)	Hormone pathways	Unigene72129_All	AT2G27150.1	54.188	TraesCS7D02G498100.1	99.457	chr7D:604889375-604903648
PYL2	Hormone pathways	CL10823.Contig1_All	AT2G26040.1	62.147	TraesCS7D02G364600.1	98.98	chr7D:470837323-470838275
PP2C59	Hormone pathways	CL11032.Contig3_All	AT4G31750.1	76.451	TraesCS7A02G423900.1	99.336	chr7A:616747780-616750237
PP2C68	Hormone pathways	Unigene22106_All	AT2G29380.1	53.444	TraesCS5A02G183600.1	100	chr5A:383418336-383419385
PP2C68	Hormone pathways	Unigene18381_All	AT2G29380.1	53.444	TraesCS5A02G183600.1	99.713	chr5A:383418336-383419385
PP2C68	Hormone pathways	Unigene28725_All	AT2G29380.1	53.444	TraesCS5A02G183600.1	100	chr5A:383418336-383419385
SnRK2.5	Hormone pathways	Unigene67857_All	AT5G63650.1	74.841	TraesCS1B02G281100.1	99.034	chr1B:489249385-489253102
CIPK23	Hormone pathways	Unigene23141_All	AT1G30270.1	80.751	TraesCS2B02G271700.1	100	chr2B:373301321-373305756
CIPK19	Hormone pathways	Unigene30394_All	AT4G18700.1	65.948	TraesCS1A02G333800.1	100	chr1A:521629027-521630592
SnRK2.1	Hormone pathways	Unigene41804_All	AT1G78290.2	73.52	TraesCS2A02G493800.1	99.312	chr2A:726029783-726034011
CIPK29	Hormone pathways	Unigene47446_All	AT2G30360.1	44.235	TraesCS2A02G106500.1	98.152	chr2A:58548059-58550982
CIPK29	Hormone pathways	Unigene49646_All	AT2G30360.1	45.232	TraesCS2B02G124100.1	97.018	chr2B:92489631-92490941
SARD1	Hormone pathways	Unigene41545_All	AT5G57580.1	45.093	TraesCS3D02G041800.1	96.644	chr3D:16032112-16034372
UDP-glucosylt ransferase	Hormone pathways	Unigene38116_All	AT1G05675.1	42.702	TraesCS2D02G055000.1	93.08	chr2D:22144303-22146318
TGA1	Hormone pathways	CL19267.Contig2_All	AT5G65210.1	62.236	TraesCS3A02G372400.1	93.617	chr3A:623047081-623054386
PR5	Hormone pathways	Unigene146_All	AT5G38280.1	34.958	TraesCS2D02G419800.1	90.397	chr2D:533855971-533858548

PR3	Hormone pathways	Unigene42382_All	AT3G12500.1	73.869	TraesCS2D02G033100.1	98.515	chr2D:12875568-12877603
PR3	Hormone pathways	Unigene47824_All	AT3G12500.1	71.839	TraesCSU02G169600.1	81.215	chrUn:249445982-249447623
COI1	Hormone pathways	Unigene70705_All	AT2G39940.1	72.839	TraesCS1A02G279100.1	100	chr1A:474699335-474703163
jasmonate ZIM domain	Hormone pathways	Unigene61572_All	AT5G13220.3	33.673	TraesCS2D02G285300.1	100	chr2D:361392674-361395409
ethylene receptor-2	Hormone pathways	Unigene14833_All	AT3G23150.2	53.968	TraesCS2A02G000200.1	91.781	chr2A:258830-262973
nitrate reductase	Metabolic signaling pathways	Unigene38911_All	AT1G37130.1	69.111	TraesCS6B02G024900.1	70.286	chr6B:15128191-15134280
glutamine synthetase	Metabolic signaling pathways	CL19806.Contig9_All	AT1G66200.1	85.112	TraesCS6B02G327500.1	100	chr6B:577183711-577187787
Glutamate dehydrogenase	Metabolic signaling pathways	Unigene20952_All	AT5G18170.1	84.672	TraesCS5D02G442000.1	99.757	chr5D:494216160-494219691
ATP-dependent 6-phosphofructokinase 5, chloroplastic-like	Metabolic signaling pathways	Unigene50369_All	AT2G22480.1	66.602	TraesCS7D02G083200.1	99.432	chr7D:49883366-49889314
pyruvate kinase	Metabolic signaling pathways	Unigene65204_All	AT4G26390.1	79.108	TraesCS4B02G174700.1	41.322	chr4B:383095812-383099928
pyruvate dehydrogenase E1 alpha subunit, mitochondrial precursor	Metabolic signaling pathways	Unigene1910_All	AT1G24180.1	79.692	TraesCS6B02G342000.1	51.913	chr6B:601880291-601884370
aldehyde dehydrogenase family 3 member F1-like	Metabolic signaling pathways	Unigene32875_All	AT4G36250.1	51.891	TraesCSU02G066700.1	93.038	chrUn:51895598-51903317
galacturonosyl transferase	Metabolic signaling	Unigene59889_All	AT4G38270.2	60.263	TraesCSU02G105800.1	100	chrUn:92738495-92742079

	pathways						
galacturonosyl transferase	Metabolic signaling pathways	Unigene72853_All	AT4G38270.2	76.667	TraesCSU02G105800.1	100	chrUn:92738495-92742079
galacturonosyl transferase	Metabolic signaling pathways	Unigene74389_All	AT4G38270.2	77.667	TraesCSU02G105800.1	100	chrUn:92738495-92742079
pectinesterase	Metabolic signaling pathways	Unigene63411_All	AT3G24130.1	45.246	TraesCS7A02G120400.1	80	chr7A:77530319-77532414
UDP-glucuronate 4-epimerase 6-like	Metabolic signaling pathways	Unigene53686_All	AT3G23820.1	64.114	TraesCS7B02G153900.1	100	chr7B:204685214-204687519
UDP-glucose 6-dehydrogenase	Metabolic signaling pathways	Unigene43656_All	AT3G29360.2	90	TraesCS5D02G404300.1	87.578	chr5D:469501070-469503575
beta-glucosidase	Metabolic signaling pathways	Unigene28759_All	AT2G44480.5	55.414	TraesCS3D02G440600.1	81.013	chr3D:551200844-551204713
beta-glucosidase	Metabolic signaling pathways	Unigene59325_All	AT2G44480.5	55.414	TraesCS3D02G440600.1	82.013	chr3D:551200844-551204713
trehalose 7-phosphate phosphatase	Metabolic signaling pathways	Unigene46665_All	AT4G12430.1	61.008	TraesCS1D02G213700.1	48.718	chr1D:298692275-298696295
NAD-dependent malic enzyme	Metabolic signaling pathways	Unigene17152_All	AT2G13560.1	85.714	TraesCS2A02G237000.1	91.071	chr2A:306158186-306177284
NAD-dependent malic enzyme	Metabolic signaling pathways	Unigene28986_All	AT2G13560.1	86.792	TraesCS2A02G237000.1	92.453	chr2A:306158186-306177284
CYPPDKZM2	Metabolic signaling pathways	Unigene75092_All	AT4G15530.2	84.314	TraesCS1A02G253200.1	98.305	chr1A:444768026-444776770
transketolase	Metabolic signaling pathways	Unigene2526_All	AT3G60750.1	85.174	TraesCS7D02G066500.1	85.174	chr7D:37285159-37288724
leucyl aminopeptidase	Metabolic signaling pathways	Unigene46510_All	AT5G13940.1	72.746	TraesCS5D02G122300.1	56.936	chr5D:176092281-176109994

psaA	Metabolic signaling pathways	Unigene5769_All	ATCG00350.1	95.867	TraesCS5D02G010900.1	95.867	chr5D:6244418-6246670
F-type H+-transporting ATPase	Metabolic signaling pathways	Unigene75325_All	AT4G09650.1	50.575	TraesCS6A02G303800.1	50.575	chr6A:536770705-536771608
glyceraldehyde-3-phosphate dehydrogenase	Metabolic signaling pathways	Unigene36189_All	AT3G04120.1	85.152	TraesCS6D02G196300.1	90	chr6D:272639825-272642873
tetraspanin-19	Metabolic signaling pathways	Unigene45393_All	AT2G20740.2	46.544	TraesCS2A02G587000.1	98.148	chr2A:777289753-777292597
tetraspanin-19	Metabolic signaling pathways	Unigene31417_All	AT2G20740.2	46.875	TraesCS2B02G596400.1	97.561	chr2B:779850560-779853570
chlorophyll a-b binding protein of LHCII type 1	Metabolic signaling pathways	Unigene25000_All	AT2G34420.1	87.266	TraesCS1D02G411300.1	94.361	chr1D:472560802-472562069
chlorophyll a-b binding protein of LHCII type 1	Metabolic signaling pathways	Unigene11566_All	AT2G34420.1	87.64	TraesCS1B02G432700.1	90.943	chr1B:657271989-657273125
chlorophyll a-b binding protein of LHCII type 1	Metabolic signaling pathways	Unigene19155_All	AT2G34420.1	87.218	TraesCS5D02G357600.1	98.868	chr5D:438359980-438360777
receptor-like protein kinase 5	Network of CMPG1-V module	Unigene33096_All	AT5G25930.1	45.339	TraesCS1D02G344700.1	82.671	chr1D:433213314-433216933
Serine-threonine kinase receptor	Network of CMPG1-V module	CL4316.Contig5_All	AT3G15610.1	80.882	TraesCS5A02G189200.1	99.118	chr5A:391548711-391554182
hexokinase	Network of CMPG1-V module	CL20093.Contig21_All	AT5G25930.1	42.296	TraesCS3D02G276200.1	87.396	chr3D:383109644-383113441
interleukin-1 receptor-associated kinase	Network of CMPG1-V module	Unigene27649_All	AT2G02800.1	70.69	TraesCS4D02G286100.1	100	chr4D:456493885-456497502
serine/threoni	Network of	Unigene40477_All	AT2G05940.1	68.405	TraesCS7D02G165300.1	98.214	chr7D:115047816-115049985

ne-protein kinase	CMPG1-V module						
serine/threonine-protein kinase SD1-29	Network of CMPG1-V module	CL8366.Contig6_All	AT4G21390.2	43.581	TraesCS2D02G217400.1	99.003	chr2D:180926044-180934087
Protein kinase APK1A	Network of CMPG1-V module	Unigene23414_All	AT2G05940.1	68.405	TraesCS7A02G163300.1	86.798	chr7A:119701737-119704345
Serine/threonine-protein kinase PBS1	Network of CMPG1-V module	CL637.Contig1_All	AT3G59360.1	75.434	TraesCS2A02G348300.1	100	chr2A:587151664-587163420
receptor-like protein kinase FERONIA	Network of CMPG1-V module	CL20266.Contig1_All	AT3G51550.1	61.64	TraesCS5D02G336300.1	98.382	chr5D:425837917-425842000
inactive receptor kinase	Network of CMPG1-V module	CL3809.Contig2_All	AT1G48480.1	52.439	TraesCS4A02G267300.1	99.257	chr4A:579609312-579612899
salicylic acid glucosyltransferase	Network of CMPG1-V module	Unigene21554_All	AT1G05675.1	42.605	TraesCS5A02G149600.1	89.165	chr5A:327573796-327581013
hydroquinone glucosyltransferase-like	Network of CMPG1-V module	Unigene10787_All	AT4G01070.1	32.623	TraesCS7A02G216000.1	99.358	chr7A:182561465-182562874
crocetin glucosyltransferase	Network of CMPG1-V module	CL18448.Contig2_All	AT4G15550.1	39.877	TraesCS3D02G120200.1	87.605	chr3D:75946541-75948546
cyanohydrin beta-glucosyltransferase	Network of CMPG1-V module	Unigene44649_All	AT1G22360.1	52.675	TraesCS5D02G324600.1	95.464	chr5D:416257321-416259242
anthocyanidin 5, 3-O-glucosyltransferase	Network of CMPG1-V module	Unigene43394_All	AT3G16520.3	44.211	TraesCS2B02G012000.1	97.021	chr2B:6178676-6180145
protein O-GlcNAc transferase	Network of CMPG1-V module	Unigene16241_All	AT3G18170.1	44.216	TraesCS1A02G351900.1	98.285	chr1A:536353547-536356836
protein O-GlcNAc transferase	Network of CMPG1-V module	Unigene3983_All	AT3G18170.1	44.473	TraesCS1D02G358500.1	98.799	chr1D:442329733-442333663
WRKY transcription	Network of CMPG1-V	Unigene74004_All	AT5G26170.1	81.429	TraesCS3A02G209800.1	91.071	chr3A:374445928-374447068

factor 2	module						
WRKY transcription factor 2	Network of CMPG1-V module	CL6784.Contig3_All	AT5G56270.1	41.278	TraesCS5B02G257300.1	99.414	chr5B:439723270-439727743
WRKY transcription factor 33	Network of CMPG1-V module	Unigene8084_All	AT2G38470.1	49.148	TraesCS1D02G292700.1	96.271	chr1D:391860635-391863613
Peroxidase 55 precursor	Network of CMPG1-V module	Unigene24158_All	AT5G14130.1	57.827	TraesCS5B02G287600.1	96.33	chr5B:473637610-473639814
peroxidase	Network of CMPG1-V module	Unigene70614_All	AT4G39720.1	68.182	TraesCS7B02G381400.1	65.541	chr7B:646209569-646210468
peroxidase	Network of CMPG1-V module	CL3881.Contig4_All	AT3G14180.1	33.159	TraesCS1B02G420500.1	97.759	chr1B:643559886-643568351

Table S4. The name, sequence of the primer pairs for qRT-PCR

**Primer name**	**Primers sequence (5'-3')**	**Application**
PR1-F	CTGGAGCACGAAGCTGCAG	qRT-PCR Primers for *TaPR1*
PR1-R	CGAGTGCTGGAGCTTGCAGT	
TGA1-F	GGGGTTTTCGTTCGTCTGAG	qRT-PCR Primers for *TaTGA1*
TGA1-R	GCCACGTTTCCTGAAGATCC	
PP2C-F	GGGCTAAAGATGACGACTGC	qRT-PCR Primers for *TaPP2C*
PP2C-R	GTGGATCCATCACCTCCATC	
COI1-F	GAGCTTCTCGCCAAGAATTG	qRT-PCR Primers for *TaCOI1*
COI1-R	TCATTTGTCCCCATGAAGGT	
ETR1-F	CGTACGACATGAACCCCTTC	qRT-PCR Primers for *TaETR1*
ETR1-R	GAAATTGGCGATCCTCTTGA	
NR-F	GCATGATCAACGAGCTCAAC	qRT-PCR Primers for *TaNR*
NR-R	CAGAAGCACCAGCACCAGTA	
GS-F	AGGATCCGGTATCGACATCA	qRT-PCR Primers for *TaGS*
GS-R	CATTGTGCCTCTTGTTGGTG	
LHCII-F	CGCAAAGTATCCTTGCCATT	qRT-PCR Primers for *TaLHCII*
LHCII-R	AAGAAGCCGAACATGGAGAA	
NADME-F	TGGGATTGGTGTTGTGAATG	qRT-PCR Primers for *TaNAD-ME*
NADME-R	GGACGTGCATCTGGGTCTAT	
TSPAN-F	AATCGCCGCTGATGTATTTC	qRT-PCR Primers for *TaTSPAN*
TSPAN-R	TCGCAAGAATGATTGACAGC	
pfkA-F	AAGAGAGAGCACCCCACTGA	qRT-PCR Primers for *TapfkA*
pfkA-R	GCCAGTAAACCCAGCCATTA	
ALDH-F	CGCAATCTACGCTTTCACAA	qRT-PCR Primers for *TaALDH*
ALDH-R	GGCTGAAAAGCTCAAAGGTG	
GLUC-F	CGTTGGCCTTATCCTTTGAC	qRT-PCR Primers for *TaGLUC*
GLUC-R	GGATGGAAGACGAGAACCAA	
TPP-F	CGCCTATTGTGGACAATCCT	qRT-PCR Primers for *TaTPP*
TPP-R	TGGTGACCATTGGACTGAGA	
Tubulin-F	GATGCAGCCAACAACTTCGCC	qRT-PCR Primers for *Tubulin*
Tubulin-R	CAGTTCCACCTCCAACAGCGT	
SnRK2.1-F	TTAGCGAGGATGAGGGAA	qRT-PCR Primers for *TaSnRK2.1*
SnRK2.1-R	CGACAGTTGACTTCGGTTGA	
SnRK2.5-F	TCTGCGACTTCGGATACTCA	qRT-PCR Primers for *TaSnRK2.5*
SnRK2.5-R	AAGTTCTTGGGGTCCTTGGT	
CIPK23-F	CGCTGTCGACTACTGCCATA	qRT-PCR Primers for *TaCIPK23*
CIPK23-R	ATTGGGAGTTCCACAAGTCG	
