# Supplementary figures and images for: The Regulatory Network of CMPG1-V in Wheat–Blumeria graminis f. sp. tritici Interaction Revealed by Temporal Profiling Using RNA-Seq
Source: Int J Mol Sci. 2020 Aug 19;21(17):5967. doi: 10.3390/ijms21175967 (PMC7504233; doi:10.3390/ijms21175967)

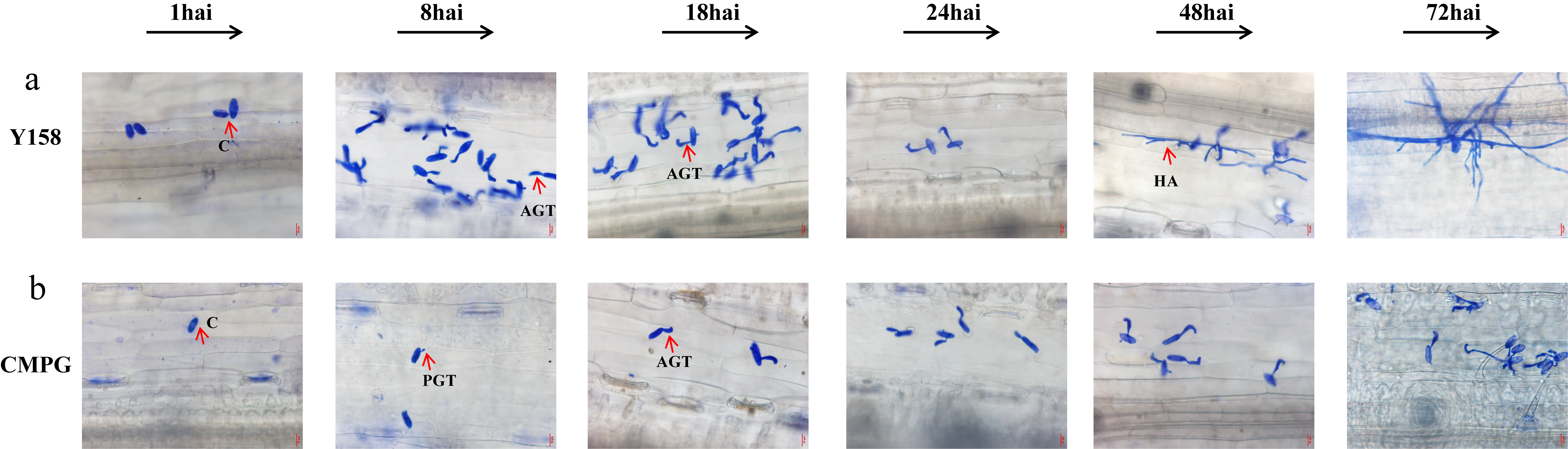

Supplement: Supplementary file 1 [file ijms-21-05967-s001.zip › Supplementary/Supplementary-figure/Supplementary FigureS1.tif]

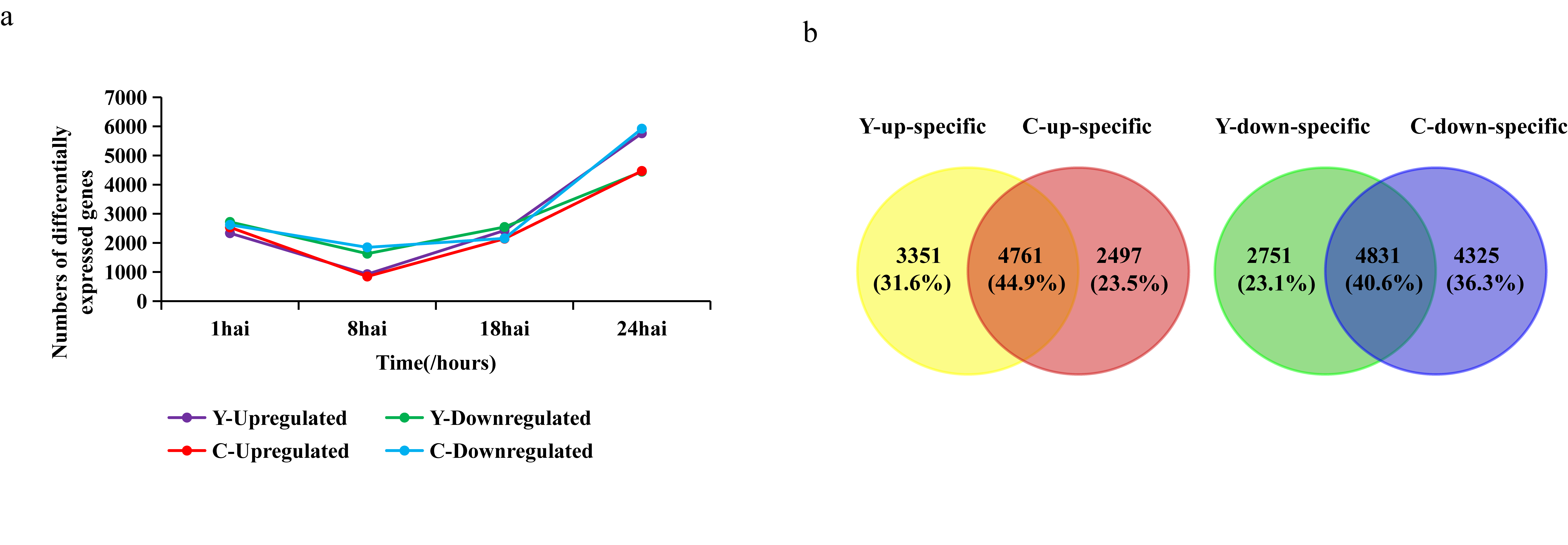

Supplement: Supplementary file 1 [file ijms-21-05967-s001.zip › Supplementary/Supplementary-figure/Supplementary FigureS2.tif]

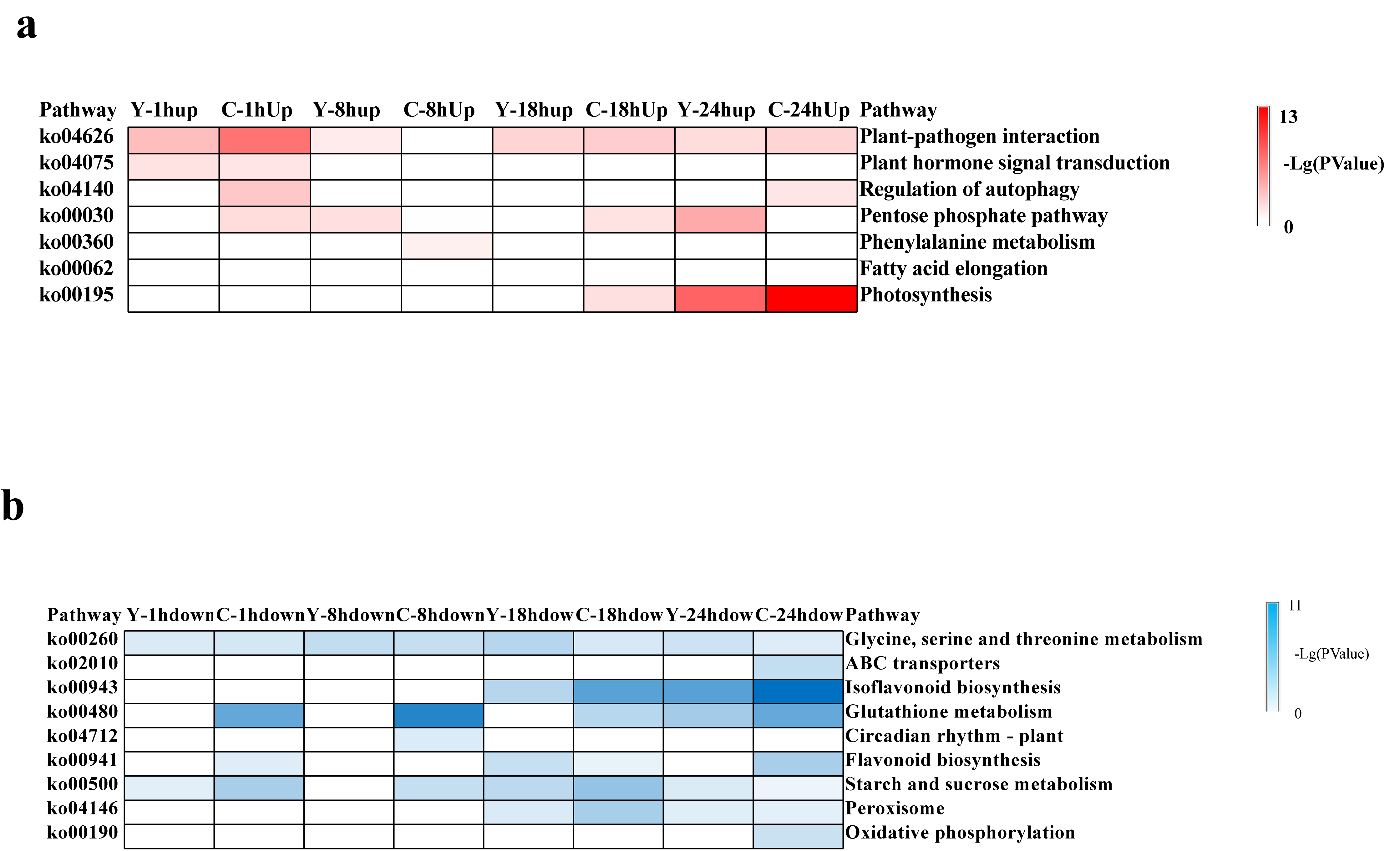

Supplement: Supplementary file 1 [file ijms-21-05967-s001.zip › Supplementary/Supplementary-figure/Supplementary FigureS6.tif]

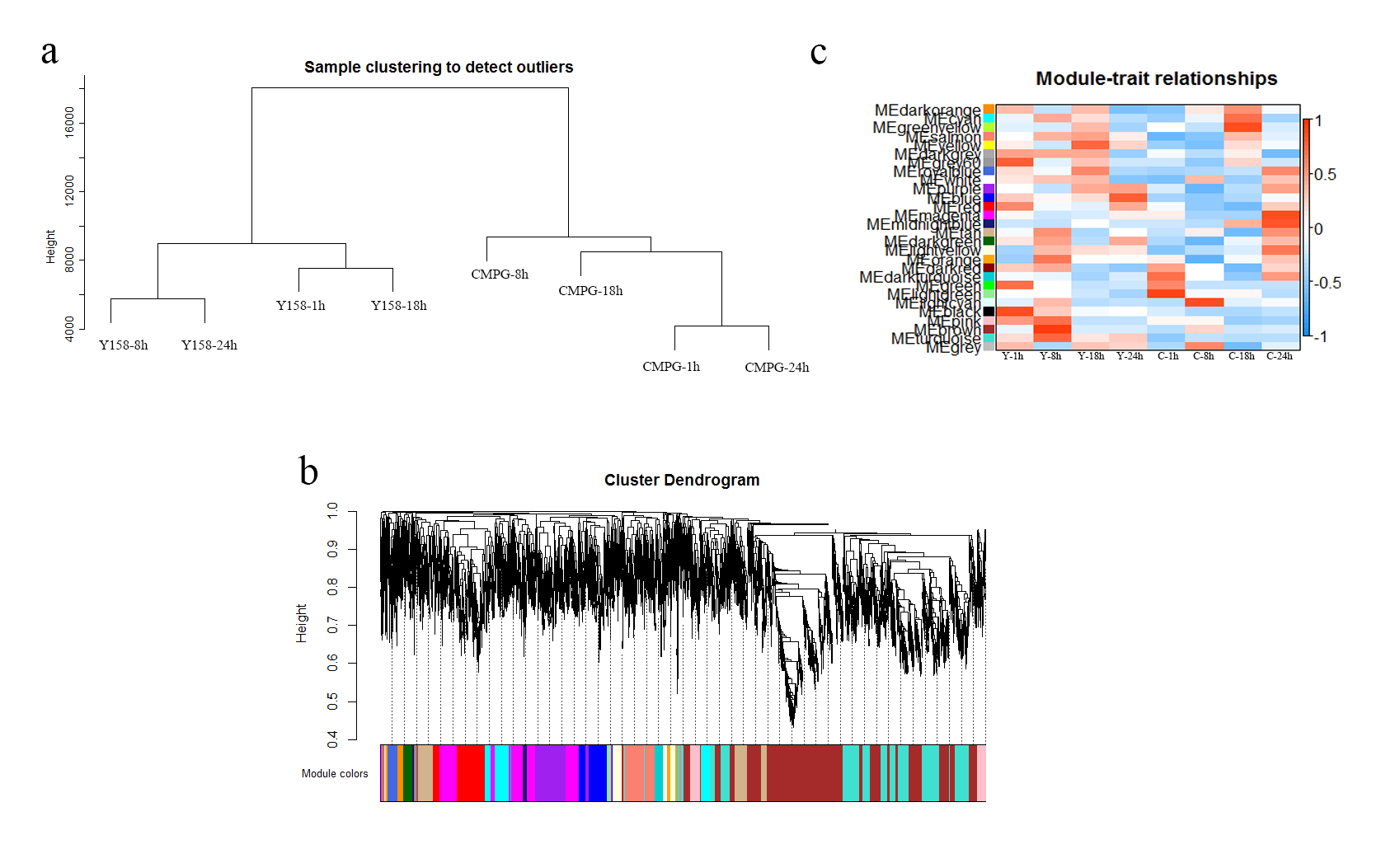

Supplement: Supplementary file 1 [file ijms-21-05967-s001.zip › Supplementary/Supplementary-figure/Supplementary FigureS9.tif]
